# Factors influencing self-harm thoughts and behaviours over the first year of the COVID-19 pandemic in the UK: longitudinal analysis of 49 324 adults

**DOI:** 10.1192/bjp.2021.130

**Published:** 2022-01

**Authors:** Elise Paul, Daisy Fancourt

**Affiliations:** Department of Behavioural Science and Health, University College London, UK

**Keywords:** Self-harm behaviours, longitudinal studies, COVID-19, self-harm thoughts, adversity

## Abstract

**Background:**

There is concern that the COVID-19 pandemic and its aftermath will result in excess suicides by increasing known risk factors such as self-harm, but evidence on how pandemic-related risk factors contribute to changes in these outcomes is lacking.

**Aims:**

To examine how different COVID-19-related experiences of and worries about adversity contribute to changes in self-harm thoughts and behaviours.

**Method:**

Data from 49 324 UK adults in the University College London COVID-19 Social Study were analysed (1 April 2020 to 17 May 2021). Fixed-effects regressions explored associations between weekly within-person variation in five categories of adversity experience and adversity worries with changes in self-harm thoughts and behaviours across age groups (18–29, 30–44, 45–59 and 60+ years).

**Results:**

In total, 26.1% and 7.9% of respondents reported self-harm thoughts and behaviours respectively at least once over the study period. The number of adverse experiences was more strongly related to outcomes than the number of worries. The largest specific adversity contributing to increases in both outcomes was having experienced physical or psychological abuse. Financial worries increased the likelihood of both outcomes in most age groups, and having had COVID-19 increased the likelihood of both outcomes in young (18–29 years) and middle-aged (45–59 years) adults.

**Conclusions:**

Findings suggest that a significant portion of UK adults may be at increased risk for self-harm thoughts and behaviours during the pandemic. Given the likelihood that the economic and social consequences of the pandemic will accumulate, policy makers can begin adapting evidence-based suicide prevention strategies and other social policies to help mitigate its consequences.

Numerous studies have shown that the COVID-19 pandemic is having a detrimental impact on population mental health, and although not inevitable, there are concerns that suicide rates will subsequently increase.^1^ Although other high-income countries have reported either no meaningful change or a decrease in suicide rates in the first months of the pandemic,^2^ temporarily lowered suicide rates have been observed in the early phases of other crises, such as natural disasters and epidemics, that were then followed by increases.^3^ This pattern has already been observed during the COVID-19 pandemic in Japan, where the first 5 months were marked by a 14% reduction in suicides, followed by a 16% increase in overall suicides, with a 49% increase among children and adolescents and a 37% increase among females.^4^ There are several reasons why suicides may increase once the immediate crisis has passed. First, the COVID-19 pandemic has involved the exacerbation of known risk factors for suicide such as unemployment, mental health problems, intimate partner violence and insufficient access to mental healthcare that may not immediately resolve as the pandemic abates.^1,5^ Second, the cumulative effects of lockdowns, job losses and uncertainty during the pandemic itself may start to take a toll over time.^1,5^ Third, the International Monetary Fund predicts that the global recession resulting from the COVID-19 pandemic will be the worst since the Great Depression,^6^ and research has consistently identified links between economic recessions, large-scale unemployment and increases in suicide rates.^7^ All of these stressful circumstances and life events have the potential to increase risk for suicide through increasing mental health difficulties such as depression, defeat, anxiety and a sense of entrapment.^8,9^

One reason for concern about a potential future increase in suicide deaths as a result of the pandemic is that there is already evidence that risk factors for death by suicide have been increasing. Thinking about self-harming, suicide or death and intentionally damaging or injuring oneself have been widely observed to be risk factors for death by suicide.^10,11^ A number of studies have suggested that prevalence rates for thinking about self-harm or suicide or engaging in self-harming have been higher during the pandemic than previously.^12–14^ Although clinical presentations for self-harm were significantly lower in the early months of the COVID-19 pandemic compared with prior trends,^15^ this could have been due to fears of contracting COVID-19 in hospitals and not wanting to be a burden on the healthcare system.^5,16^ Even pre-pandemic, the majority of individuals who self-harm or consider suicide do not seek help from clinical services.^17^

In considering why the above-mentioned risk factors for suicide (e.g. unemployment, mental health difficulties and domestic abuse) may have increased in the first months of the current pandemic, several studies have identified potential predictors. Financial strain,^18^ experiencing physical/psychological abuse^12^ and receiving a COVID-19 diagnosis,^12,18^ legal problems, ongoing arguments with a partner and worries about a life-threatening illness or injury in a family member or close friend have been associated with thinking about and carrying out self-harm,^19^ as have new and exacerbated mental health problems and insufficient access to mental healthcare.^20^ However, studies exploring predictors of self-harm thoughts and behaviours have been limited in which predictors they have considered and considering predictors at a single moment in time. As the social and economic circumstances of the pandemic are changing so fast, predictors identified early on in the pandemic may no longer be relevant. So, it is important to have updated information on what is causing people to think about harming themselves and to actually do so as the pandemic continues. Finally, it is important to identify which factors are associated over time not just with an increased overall risk in self-harm thoughts and behaviours but also with dynamic changes (both increases and decreases) so that modifiable targets to reduce self-harm can become the subject of future interventions.

Therefore, the aim of this study is to establish which factors are associated with changes over time in thoughts of death or self-harm (hereafter referred to as ‘self-harm thoughts’) and self-harm behaviours in a large sample of UK adults across the first 59 weeks of the COVID-19 pandemic. Specifically, we explore the time-varying longitudinal relationships between (a) adverse experiences and (b) worries about adverse experiences on the one hand and changes in self-harm thoughts and behaviours on the other, and how these associations vary by age. Identifying specific concerns and adversities that are risk factors for self-harm thoughts and behaviours will provide an opportunity for policymakers to address those issues by designing policies to mitigate the impact of the COVID-19 pandemic and the anticipated upcoming economic recession.

## Method

### Study design and participants

Data were drawn from the University College London (UCL) COVID-19 Social Study, a large panel study of the psychological and social experiences of over 75 000 adults (aged 18+) in the UK during the COVID-19 pandemic. The study commenced on 21 March 2020 and involves online weekly data collection for the first 22 weeks of the COVID-19 lockdown in the UK (until 15 August 2020), then monthly collection thereafter. Sampling was not random and therefore is not representative of the UK population, but the sample is heterogeneous. More information on sampling methods can be found in the supplementary material available at https://doi.org/10.1192/bjp.2021.130.

The authors assert that all procedures contributing to this work comply with the ethical standards of the relevant national and institutional committees on human experimentation and with the Helsinki Declaration of 1975, as revised in 2008. All procedures involving human participants were approved by the UCL Ethics Committee (approval number: 12467/005). Written informed consent was obtained from all participants.

As questions asked about adverse experiences and worries about adversity in the previous week, we focused on data collected from 1 April 2020 (1 week after lockdown commenced in the UK) to 17 May 2021 (66 308 participants; 918 440 observations). We then limited our analysis to participants who had taken part on three or more occasions during this period (52 569 participants; 899 447 observations). We further excluded participants who had missing data on any study variable for at least three interviews (*n* = 3245). This resulted in the final sample of 49 324 participants totalling 849 452 observations (see supplementary Table 1 for descriptive characteristics of excluded and included participants).

### Outcomes: self-harm thoughts and behaviours

Self-harm thoughts were measured with an item from the nine-item Patient Health Questionnaire (PHQ-9)^21^ and self-harm behaviours were measured with a similar study-developed item (see supplementary material). Responses to both items were collapsed into presence (one or two days, more than half the days, or nearly every day) or absence (not at all) at each time point.

### Exposures

#### Adversity experiences

Five categories of adversity measured weekly for the first 22 weeks of the study (1 April to 21 August 2020) and then monthly to 17 May 2021 were considered: financial adversity, COVID-19 illness, family/friend illness or bereavement, experiencing physical or psychological abuse and not being able to access essential items. Each category of adversity was treated as binary (absent versus present). More detailed description of these measures can be found in the supplementary material.

#### Worries about adversity

Worries about adverse experiences were measured at the same time as the adversity measures and selected to correspond to these variables. Each category of worry was operationalised as binary (absent versus present): financial worries, COVID-19 illness, social and relationship worries, concerns about safety and security, and worries about accessing essentials. See the supplementary material for further description of these measures.

### Statistical analysis

First, we describe weekly patterns in our outcome, adversity and worries about adversity variables from 1 April 2020 through 17 May 2021. We then use fixed-effects regression to analyse the time-varying associations between changes in both experiences of and worries about adversity and changes in self-harm thoughts and behaviours across these 59 weeks. In this approach, individuals serve as their own reference point, which accounts for any confounding associations between time-invariant (stable) covariates such as socioeconomic status, genetics, personality and history of mental illness between predictors and outcomes.^22^ Our analyses consisted of regressing each outcome measure on (a) the total number of adversity experiences and adversity worries jointly and (b) individual categories of adversity and worry about adversity for the total sample and then stratified by age. All regression models adjusted for day of week (categorical) and days since lockdown commenced (continuous). Resulting regression coefficients were exponentiated and presented as odds ratios along with 95% confidence intervals. See supplementary material for more detail, including the basic model equation.

Sensitivity analyses included: models that included continuous measures of (a) weekly depression symptoms, (b) weekly anxiety symptoms and (c) the physical/psychological abuse variable separated into physical abuse and psychological abuse. To increase representativeness of the UK general population, data were weighted to the proportions of gender, age, ethnicity, country and education in the UK; weights were constructed using the ebalance programme in Stata^23^ based on data obtained from the Office for National Statistics.^24^ Analyses were conducted using Stata version 16 for Windows.^25^

## Results

### Sample characteristics

In the unweighted analytic samples of participants with any change in self-harm thoughts (*n* = 11 580) or self-harm behaviours (*n* = 3747) during the study period, women (78% in both samples) and individuals with a university degree or higher (69% in the self-harm thoughts sample; 64% in the self-harm behaviours sample) were overrepresented (supplementary Table 2). In contrast, people from ethnic minority groups (6% in both samples) and young adults (aged 18–29; 10% in the self-harm thoughts sample; 14% in the self-harm behaviours sample) were underrepresented. After weighting, the samples reflected population proportions of these demographic characteristics (e.g. 55% women in both samples; people with a university degree or above: 36% in the self-harm thoughts sample and 30% in the self-harm behaviours sample; and ethnic minorities: 12% and 13% in the self-harm thoughts and self-harm behaviours samples respectively).

The average proportions of the sample reporting self-harm thoughts and self-harm behaviours over the first 59 weeks of the pandemic were relatively stable from the beginning of the pandemic to early autumn (supplementary Fig. 1(a)), but fluctuations were then seen in both outcomes in September and October 2020. The average total number of worries about adversity was consistently about three times higher than actual adversity experiences across the 59 weeks, with fluctuations seen starting in September 2020 (supplementary Fig. 1(b)), when data collection switched from weekly to monthly.

Over a quarter (26.1%) of respondents in the total sample reported having self-harm thoughts at least once over the first 59 weeks of the pandemic (supplementary Table 3), and nearly 1 in 10 (7.9%) had self-harmed at least once (supplementary Table 4). There was within-individual variation over time in self-harm thoughts and behaviours outcome measures in 11 580 and 3747 individuals respectively, suggesting that fixed effects was a valid approach. Descriptive statistics for predictor and outcome variables for each for these two samples are presented in supplementary Table 5.

### Associations between total number of adversities and worries and self-harm thoughts and behaviours

Each additional adverse event experienced was associated with a 1.56 (95% CI 1.52–1.60) times higher odds of self-harm thoughts (Table 1 and supplementary Fig. 2) and each additional adversity was associated with a nearly two-fold (OR = 1.80, 95% CI 1.72–1.87) increased likelihood of self-harm behaviours (Table 2 and supplementary Fig. 3) in the total sample. Increased likelihood of both outcomes in the total sample was smaller for the total number of worries about adversity (self-harm thoughts: OR = 1.28, 95% CI 1.26–1.30; self-harm behaviours: OR = 1.16, 95% CI 1.13–1.19) than actual adversity experiences. This also applied to age-stratified analyses: the total number of adversity experiences (self-harm thoughts: OR = 1.43–1.61; self-harm behaviours: OR = 1.68–2.02) was more strongly associated with both outcomes than adversity worries in each of the four age groups (self-harm thoughts: OR = 1.19–1.33; self-harm behaviours: OR = 1.07–1.26).

**Table 1:** Fixed-effects logistic regression models predicting within-individual change in self-harm thoughts from the total number of adversity experiences and adversity worries^a^

Variable	Self-harm thoughts														
	Total sample			Ages 18–29			Ages 30–44			Ages 45–59			Ages 60+		
	OR	95% CI lower	95% CI upper	OR	95% CI lower	95% CI upper	OR	95% CI lower	95% CI upper	OR	95% CI lower	95% CI upper	OR	95% CI lower	95% CI upper
Total number of adversity experiences	1.56	1.52	1.60	1.55	1.45	1.66	1.43	1.35	1.51	1.60	1.53	1.68	1.61	1.53	1.70
Total number of worries	1.28	1.26	1.30	1.33	1.28	1.38	1.19	1.15	1.23	1.30	1.26	1.34	1.32	1.28	1.36
Observations, *n*	206 714			16 495			53 752			74 635			61 832		
Participants, *n*	11 580			1168			3394			4069			2949		

a. Adversity experiences (0–5) and worries (0–5) variables are weighted to the proportions of gender, age, ethnicity, country and education obtained from the Office for National Statistics. Individual adversity experiences and worries variables are binary. Analyses were further adjusted for day of the week and time since lockdown began.

**Table 2:** Fixed-effects logistic regression models predicting within-individual change in self-harm behaviours from the total number of adversity experiences and adversity worries^a^

Variable	Self-harm behaviours														
	Total sample			Ages 18–29			Ages 30–44			Ages 45–59			Ages 60+		
	OR	95% CI lower	95% CI upper	OR	95% CI lower	95% CI upper	OR	95% CI lower	95% CI upper	OR	95% CI lower	95% CI upper	OR	95% CI lower	95% CI upper
Total number of adversity experiences	1.80	1.72	1.87	1.68	1.54	1.83	1.70	1.55	1.86	1.79	1.67	1.93	2.02	1.85	2.21
Total number of worries	1.16	1.13	1.19	1.26	1.19	1.34	1.07	1.01	1.14	1.19	1.13	1.25	1.10	1.04	1.17
Observations, *n*	63 767			7 687			18 594			23 859			13 627		
Participants, *n*	3747			530			1201			1329			687		

a. Adversity experiences (0–5) and worries (0–5) variables are weighted to the proportions of gender, age, ethnicity, country and education obtained from the Office for National Statistics. Individual adversity experiences and worries variables are binary. Analyses were further adjusted for day of the week and time since lockdown began.

### Associations between individual categories of adversity and worries and self-harm thoughts and behaviours

When examining individual categories of adversity and adversity worries, having experienced psychological or physical abuse had the largest associations with both outcomes across all age groups and in the total sample (Tables 3 and 4 and supplementary Figs 4 and 5). Odds ratios were slightly higher for self-harm behaviours (OR = 3.39–6.96) than for self-harm thoughts (OR = 3.37–3.90). Increases in financial adversities and worries, social/relationship concerns and concerns about one's safety (‘threats to safety’) increased the likelihood of later self-harm thoughts in all age groups. Financial concerns generally had larger magnitudes of association (self-harm thoughts: OR = 1.35–1.72; self-harm behaviours: OR = 1.11–1.40) with both outcomes than actual adversity experiences (self-harm thoughts: OR = 1.12–1.27; self-harm behaviours: OR = 0.80–1.14). Having had COVID-19 increased the likelihood of both outcomes in young adults (aged 18–29) and in adults aged 45–59. However, concerns about becoming ill with COVID-19 increased the likelihood of self-harm thoughts only in older adults; such concerns decreased the likelihood of self-harm thoughts in the total sample, young adults and adults aged 30–44.

**Table 3:** Fixed-effects logistic regression models predicting within-individual change in self-harm thoughts from individual categories of adversity experiences and worries^a^

Variable	Self-harm thoughts														
	Total sample			Ages 18–29			Ages 30–44			Ages 45–59			Ages 60+		
	OR	95% CI lower	95% CI upper	OR	95% CI lower	95% CI upper	OR	95% CI lower	95% CI upper	OR	95% CI lower	95% CI upper	OR	95% CI lower	95% CI upper
Adversity experiences															
Financial	1.18	1.13	1.25	1.27	1.12	1.42	1.16	1.05	1.29	1.12	1.03	1.22	1.25	1.12	1.40
Accessing essentials	1.28	1.19	1.37	0.93	0.76	1.14	1.00	0.87	1.17	1.66	1.46	1.89	1.33	1.17	1.52
COVID-19 illness	1.17	1.09	1.25	1.26	1.06	1.50	1.04	0.91	1.19	1.27	1.13	1.42	1.12	0.98	1.29
Illness of others/bereavement	1.14	1.08	1.21	1.29	1.10	1.50	1.02	0.89	1.16	1.23	1.11	1.37	1.09	0.98	1.21
Physical/psychological abuse	3.55	3.37	3.75	3.73	3.19	4.34	3.90	3.47	4.39	3.48	3.17	3.82	3.37	3.07	3.70
Worries															
Financial	1.46	1.40	1.52	1.35	1.22	1.49	1.35	1.24	1.47	1.72	1.59	1.86	1.38	1.27	1.50
Accessing essentials	1.18	1.13	1.24	1.43	1.28	1.59	1.06	0.97	1.15	0.99	0.92	1.08	1.39	1.28	1.50
COVID-19 illness	0.94	0.90	0.98	0.82	0.74	0.90	0.80	0.74	0.87	0.97	0.90	1.05	1.12	1.04	1.22
Social/relationship concerns	1.62	1.56	1.69	1.92	1.73	2.14	1.67	1.54	1.82	1.62	1.50	1.75	1.44	1.33	1.55
Threats to safety	1.42	1.35	1.48	1.70	1.52	1.90	1.41	1.28	1.54	1.42	1.31	1.54	1.31	1.20	1.42
Observations, *n*	206 714			16 495			53 752			74 635			61 832		
Participants, *n*	11 580			1168			3394			4069			2949		

a. Adversity experiences and worries variables are weighted to the proportions of gender, age, ethnicity, country and education obtained from the Office for National Statistics. Individual adversity experiences and worries variables are binary. Analyses were further adjusted for day of the week and time since lockdown began.

**Table 4:** Fixed-effects logistic regression models predicting within-individual change in self-harm behaviours from individual categories of adversity experiences and worries^a^

Variable	Self-harm behaviours														
	Total sample			Ages 18–29			Ages 30–44			Ages 45–59			Ages 60+		
	OR	95% CI lower	95% CI upper	OR	95% CI lower	95% CI upper	OR	95% CI lower	95% CI upper	OR	95% CI lower	95% CI upper	OR	95% CI lower	95% CI upper
Adversity experiences															
Financial	1.01	0.93	1.11	1.01	0.85	1.21	0.80	0.66	0.97	1.10	0.95	1.27	1.14	0.93	1.41
Accessing essentials	1.18	1.06	1.31	1.10	0.89	1.36	0.88	0.70	1.12	1.36	1.14	1.64	1.43	1.14	1.79
COVID-19 illness	1.36	1.21	1.53	2.43	1.82	3.23	1.03	0.81	1.32	1.52	1.25	1.84	0.98	0.74	1.29
Illness of others/ bereavement	1.23	1.11	1.36	1.51	1.23	1.85	1.20	0.95	1.52	1.14	0.96	1.35	1.15	0.93	1.41
Physical/ psychological abuse	4.94	4.57	5.35	3.39	2.84	4.06	6.96	5.84	8.30	4.34	3.78	4.96	5.96	5.09	6.98
Worries															
Financial	1.22	1.13	1.32	1.11	0.96	1.29	1.28	1.09	1.50	1.40	1.21	1.60	1.12	0.94	1.32
Accessing essentials	1.22	1.14	1.31	1.47	1.27	1.70	1.09	0.93	1.27	1.17	1.02	1.34	1.18	1.01	1.37
COVID-19 illness	0.95	0.88	1.03	1.04	0.89	1.22	0.76	0.64	0.89	0.96	0.84	1.10	1.09	0.93	1.28
Social/relationship concerns	1.31	1.21	1.42	1.79	1.52	2.10	1.14	0.97	1.34	1.33	1.16	1.53	1.11	0.93	1.32
Threats to safety	1.25	1.16	1.35	1.12	0.95	1.31	1.70	1.44	2.01	1.26	1.10	1.43	1.11	0.95	1.30
Observations, *n*	63 767			7687			18 594			23 859			13 627		
Participants, *n*	3747			530			1201			1329			687		

a. Adversity experiences and worries variables are weighted to the proportions of gender, age, ethnicity, country and education obtained from the Office for National Statistics. Individual adversity experiences and worries variables are binary. Analyses were further adjusted for day of the week and time since lockdown began.

In the two younger age groups (18–29 and 30–44) and in older adults (aged 60+), social and relationship concerns had the second strongest associations with self-harm thoughts (OR = 1.67–1.92) after experiencing physical/psychological abuse. In adults aged 45–59, the second strongest associations with self-harm thoughts after physical/psychological abuse were for financial concerns (OR = 1.72, 95% CI 1.59–1.86).

In older adults (aged 60+), the second strongest association with self-harm behaviours was for having not been able to access essential items (OR = 1.43, 95% CI 1.14–1.79), whereas the second strongest association was for having had COVID-19 in adults aged 45–59 (OR = 1.52, 95% CI 1.25–1.84) and young adults (OR = 2.43, 95% CI 1.83–3.23) and for threats to personal safety in the 30–44 age group (OR = 1.70, 95% CI 1.44–2.01).

### Sensitivity analyses

When accounting for anxiety and depression symptoms within models, results were largely similar (supplementary Tables 6–9). Analyses examining physical abuse and psychological abuse as individual adversity experiences showed different patterns of association with outcomes for each abuse type (supplementary Tables 10 and 11).

## Discussion

Both experiencing adversities and worrying about adversities were associated with an increased likelihood of self-harm thoughts and actually engaging in self-harm behaviours across the first 59 weeks of the COVID-19 pandemic. These results were found across age groups, with strongest associations for the total number of adversity experiences compared with the number of worries about adversities. The proportion in our sample reporting self-harm thoughts (26.1%) and self-harm behaviours (7.9%) at least once over the first year of the pandemic was higher than in other population-based studies conducted in the first few months of the pandemic, when approximately 10%^13,14^ of adults reported suicidal/self-harm thoughts and around 1%^13^ reported self-harm behaviours. Our findings are, however, similar to one US study which found that 31% of adults had reported thoughts of suicide/self-harm in the past 2 weeks.^18^

### Physical/psychological abuse and financial uncertainty and adversity

The largest predictor by far of both thinking about and engaging in self-harm was experiencing physical or psychological abuse, and this finding was consistent across all four age groups examined. Sensitivity analyses suggested that physical abuse is making larger contributions than psychological abuse to self-harm behaviours, whereas the sizes of the associations of both abuse types with self-harm thoughts were more similar. A range of literature outside of pandemic circumstances shows that different forms of abuse, including domestic violence, are predictors of self-harm behaviours and suicide.^26,27^ That increases in domestic abuse would occur during stay-at-home orders was anticipated early in the pandemic,^28^ and has been demonstrated in countries internationally. For example, the number of calls to emergency domestic abuse hotlines in the European Union had increased by 60% by the end of April 2020,^29^ and intimate partner violence against women increased by 23% over the first 3 months of the first lockdown in Spain.^30^

Worsening economic circumstances have been identified as one of the causes of this increase in domestic abuse,^30^ and financial stress was another predictor of self-harm thoughts and behaviours identified by our study and by other research conducted during the current pandemic.^18^ Notably, worrying about financial adversity such as losing one's job rather than actually experiencing such an adversity was more consistently associated with self-harm thoughts and behaviours across age groups. This suggests that, thus far, economic uncertainty rather than actual adversities is having a negative impact on people's mental health, and it is possible that these associations may change in magnitude as the economic consequences of the pandemic and recession unfold. Considerable evidence indicates that economic recessions are associated with increases in rates of self-harm and suicide, particularly in the working-age population.^8^ Risk for both attempted suicide and suicide death is higher for those unemployed over the long term compared with those in shorter-term unemployment.^31,32^ However, an increase in self-harm and suicides during economic recessions is not inevitable.^1^

Lessons learned from prior economic recessions suggest multiple opportunities for how governments can respond with policies to mitigate the mental health impact of the upcoming recession.^5^ The increases in suicides that correspond to unemployment rates are not uniform across all countries but are instead modified by differential investment in social programmes to mitigate these effects.^33^ For example, following the 2008 financial crisis, an increase of 1% per capita in government spending designed to mitigate the effect of financial hardship was associated with a 0.2% decrease in suicide in Japan.^34^ In the three decades leading up to the 2008 recession, every US$10 invested per person on programmes aimed to increase chances of gainful employment resulted in a 0.04% decrease in the effect of unemployment on suicides in EU countries.^33^ Thus, our findings highlight the potential danger of the economic impacts of COVID-19 on self-harm thoughts and behaviours and, as self-harm is an important risk factor for suicide, potentially for suicide too, and suggest the importance of addressing economic concerns among individuals urgently.

### Age-related differences in associations

Although many of our findings were consistent across age groups, there were some discrepancies. For example, worrying about catching COVID-19 was associated with reduced likelihood of having self-harm thoughts and self-harm behaviours in adults aged 30–44, but this pattern was reversed in the oldest age group (aged 60+), among whom worries about falling ill were associated with increased likelihood of thoughts of harming themselves or that they would be better off dead (self-harm thoughts). This could have been influenced by public health messaging highlighting that older adults are at higher risk than younger adults for dying of the illness – a death that news coverage often portrayed as occurring alone and without the ability to say goodbye to loved ones. In contrast, having already had the illness was related to increased likelihood of both outcomes in the total sample, and this remained true in young (18–29) and middle-aged (45–59) adults for self-harm thoughts and for self-harm behaviours. It is therefore possible that the disease itself may play a role in increasing risk for self-harm thoughts and behaviours, whereas those who are particularly worried about falling ill with COVID-19 are more protective of their health and less likely to want to harm themselves. Evidence for the former, that COVID-19 illness leads to increased risk for mental health problems such as depression, self-harm thoughts and self-harm behaviours, has been documented across a range of studies.^12,35^

### Strengths and limitations

This study has a number of strengths, including the use of a large, well-stratified sample on sociodemographic groups that were weighted on the basis of population estimates of core demographics, and its longitudinal follow-up with repeated assessments of adversities, worries and self-harm thoughts and behaviours. We also used robust statistical methods to account for unobserved stable participant characteristics. However, sampling was not random and the data are therefore not representative of the general UK population. It is possible that individuals who were experiencing greater adversity and were more likely to have self-harm thoughts and behaviours were more likely to participate in the study. Nevertheless, this study did not aim to report prevalence of such experiences, but rather to identify the time-varying relationship between exposures and self-harm thoughts and behaviours. Crucially, the sample was heterogeneous and maintained its heterogeneity over time. There may have been other relevant forms of adversity and worry not captured in the current study, which may have resulted in an overestimation of the adversities and worries we did include. Finally, fixed-effects regression does not address direction of causality. Arellano–Bond models can be used to follow up fixed-effects models to account for this lack of directionality,^36^ but because linear estimation is used, we could not utilise this approach as our outcomes were binary. However, although the relationship between worries and self-harm thoughts and behaviours may have involved some bidirectionality, there is little evidence to suggest that self-harm thoughts or behaviours increase the likelihood of individuals experiencing adversities.

### Implications

Across the COVID-19 pandemic, there are concerns about potential future increases in suicide levels. Self-harm thoughts and behaviours are important and strong predictors of future suicide risk, so identifying modifiable risk factors for self-harm that can be addressed through public health interventions during the pandemic and beyond is vital.^1,5^ Our findings suggest that increases in self-harm thoughts and behaviours across the first 59 weeks of the pandemic were related to financial uncertainty, physical or psychological abuse, concern for others, not being able to access essential items and worries about one's personal safety. Suggestions have already been made for how to adapt evidence-based suicide prevention strategies to current the pandemic.^37^ For example, it has been recommended that universal interventions to mitigate the impact of poverty and unemployment on suicide risk should be implemented.^5^ Our data suggest the importance of following such strategies to try to reduce self-harm thoughts and behaviours that have the potential to drive rising suicide rates over the coming months. The findings here also suggest the need for ongoing surveillance of how these well-established risk factors for suicide and self-harm may be exacerbated by the upcoming recession and as public health measures such as social distancing continue.

## Data Availability

The COVID-19 Social Study documentation and codebook are available for download at www.covidsocialstudy.org. Statistical code is available on request from E.P. (e.paul@ucl.ac.uk).

## Supplementary material

For supplementary material accompanying this paper visit https://doi.org/10.1192/bjp.2021.130. click here to view supplementary material
