# Supplementary material for: Factors influencing self-harm thoughts and behaviours over the first year of the COVID-19 pandemic in the UK: longitudinal analysis of 49 324 adults
Source: Br J Psychiatry. 2022 Jan;220(1):31–7. doi: 10.1192/bjp.2021.130 (PMC8958127; doi:10.1192/bjp.2021.130)
Supplement: Supplementary file 1 [file S0007125021001306sup001.docx]

Supplementary Materials

Sampling methods

Three primary recruitment approaches were used. First, convenience sampling was used, including promoting the study through existing networks and mailing lists (including large databases of adults who had previously consented to be involved in health research across the UK), print and digital media coverage, and social media. Second, more targeted recruitment was undertaken focusing on groups who were anticipated to be less likely to take part in the research via our first strategy, including (i) individuals from a low-income background, (ii) individuals with no or few educational qualifications, and (iii) individuals who were unemployed. Third, the study was promoted via partnerships with third sector organisations to vulnerable groups, including adults with pre-existing mental health conditions, older adults, carers, and people experiencing domestic violence or abuse. Recruitment was refreshed in August when participants who were lost-to-follow-up were recontacted. The study was approved by the UCL Research Ethics Committee [12467/005] and all participants gave informed consent. Participants were not compensated for participation.

Outcomes

Self-harm thoughts and behaviours

Two outcome variables were assessed weekly for the first 22 weeks of the study (1 April to 21 August 2020) and then monthly to 17 May 2021. Thoughts of death or self-harm (“self-harm thoughts”) were measured with an item from the Patient Health Questionnaire (PHQ-9)^1^, an instrument often used as a screening tool for depression in primary care practice: “Over the last week, how often have you been bothered by: Thoughts that you would be better off dead or hurting yourself in some way?”. Second, self-harm behaviours were measured with a similar study-developed item: “Over the last week, how often have you been bothered by: Self-harming or deliberately hurting yourself?”. Responses to both items were rated on a four-point scale from “not at all” to “nearly every day”. Analyses focused on binary variables indicating the presence of at least some self-harm thoughts and self-harm behaviours at each time point.

Exposure variables

Adversities

Financial adversity consisted of: (i) loss of job/been unable to do paid work; (ii) spouse/partner lost their job/was unable to do paid work; (iii) major cut in household income (e.g., due to you or your partner being furloughed/put on leave/ not receiving sufficient work); (iv) unable to pay bills/rent/mortgage; and (v) evicted/lost accommodation. Illness with COVID-19 was measured with an item asking if participants had either suspected or been diagnosed with the illness. Family/friend illness or bereavement was assessed as: (i) the participant having someone close to them ill in hospital (due to COVID-19 or another cause), and (ii) lost somebody close (due to COVID-19 or another cause). Participants indicated whether they had been “physically harmed or hurt by someone else” or “bullied, controlled, intimidated, or psychologically hurt by someone else” during the last week. Responses were rated on a four-point scale ranging from “not at all” to “nearly every day”. A response to either item indicating physical or psychological abuse was categorised as present. Finally, inability to access essential items was measured by asking if participants had been unable to access sufficient food and required medication. Total weekly adversities scores were calculated by summing each of the five binary variables (range 0-5).

Worries about adversity

Two questions asked participants to select which of a list of items had caused them (i) stress (however minor) in the past week, and (ii) significant stress (something being constantly on their mind or keeping them awake at night in the past week). Responses were classified as worries whether participants said they were causing them either minor or significant stress. Financial worries was measured with three items: (i) losing your job/unemployment, (ii) finances, and (iii) work, COVID-19 illness with two: (i) catching COVID-19 and (ii) becoming seriously ill from COVID-19, social and relationship worries with five: (i) marriage or other romantic relationship, (ii) friends or family living in your household, (iii) friends or family living outside your household, (iv) neighbours, and (v) your pet), safety and security concerns with one: your own safety/security’, and worries about access essentials with two (i) food and (ii) medication. Total weekly worries scores were computed by summing these five binary variables (range 0-5).

Variables used to describe the samples

Several socio-demographic and health factors were used to describe the two samples. All were measured when participants first joined the study. These included gender (women vs men), ethnicity (white vs ethnic minority groups), age groups (age 18-29, 30-45, 46-59, 60+) and education (up to GCSE levels, A-levels or equivalent, and university degree or above). For ethnicity, participants were asked: “What is your ethnicity?”. Answer choices were: i) Asian/Asian British - Indian, Pakistani, Bangladeshi, other, ii) Black/Black British - Caribbean, African, other, iii) Mixed race - White and Black/Black British, iv) Mixed race – other, v) White - British, Irish, other, vi) Chinese/Chinese British, vii) Middle Eastern/Middle Eastern British - Arab, Turkish, other, viii) Other ethnic group, and ix) Prefer not to say. Respondents endorsing v (White- British, Irish, other) were classified as white, whilst all other categories were classified as ethnic minority groups.

We also included two health-related factors to describe the samples: self-reported diagnosis of any long-term physical health condition (e.g., asthma or diabetes) or any disability (yes vs no), and self-reported diagnosis of any long-term mental health condition (e.g., depression, anxiety) (yes vs no). Participants were asked if they had at least one of eight long-term physical health conditions (high blood pressure, diabetes, heart disease, lung disease [e.g., asthma or COPD], cancer, another clinically-diagnosed chronic physical health condition). They were also asked if they had at least one of four long-term mental health conditions (clinically-diagnosed depression, clinically-diagnosed anxiety, another clinically-diagnosed mental health problem).

Statistical Analysis

The basic model can be expressed as follows:

Outcome_𝑖𝑡_= 𝛽_0𝑡_+ 𝛽_1_𝐸𝑖𝑘𝑡 + 𝛽2𝑊𝑖𝑘𝑡 + 𝛽3𝐷𝑡 + 𝛽4𝑁𝑡 + 𝛼𝑖 + 𝜀𝑖𝑡

where Outcome*_it_* is a measure of individual 𝑖's self-harm thoughts or self-harm behaviours at time 𝑡, E is whether an individual 𝑖 was experiencing adversity 𝑘 at time 𝑡, W is whether an individual 𝑖 was worrying about adversity 𝑘 at time 𝑡, D𝑡 is a vector of indicator variables for day, *N𝑡* is a continuous variable for days since lockdown, is unobserved time invariant confounding factors, and 𝜀 is error.

Three sets of sensitivity analyses were conducted to ensure the robustness of our results. First, models with individual categories of adversities and worries were re-run which included continuous measures of weekly depression symptoms assessed with the PHQ-9^1^, without the inclusion of the self-harm thoughts item. Second, these models were estimated again, but this time with continuous measures of weekly anxiety symptoms using the Generalized Anxiety Disorder (GAD-7) scale^2^. Third, we repeated our fixed effects models using individual categories of worry and adversity with the physical/psychological abuse variable separated into two separate variables: physical abuse and psychological abuse.

To account for the non-random nature of the sample and increase representativeness of the UK general population, all data were weighted to the proportions of gender, age, ethnicity, country, and education obtained from the Office for National Statistics^3^. Weights were constructed using a multivariate reweighting method using the Stata user written command ‘ebalance’^4^. Analyses were conducted using Stata version 16^5^ and fixed effects model results plotted with the user-written command ‘coefplot’^6^. Full details on sampling, recruitment, data collection, data cleaning, weighting and sample demographics are available at <https://github.com/UCL-BSH/CSSUserGuide>.

Results

Sensitivity analyses

When accounting for anxiety and depression symptoms within models, results were largely similar. But financial adversities were no longer increased risk for either outcome in any age group and were related to decreased likelihood of outcomes in some age groups (Supplemental Tables S6-S9). Worries about financial adversity, however, remained associated with increased likelihood of both outcomes in the total sample and across some age groups. Analyses examining physical abuse and psychological abuse as individual adversity experiences showed different patterns of association for each abuse type with outcomes (Supplemental Tables S10 and S11). Whilst physical abuse (OR range = 5.77 to 19.53) had much larger associations than psychological abuse (OR range = 1.74 to 2.69) with self-harm behaviours in all age groups, the associations for these two abuse types were more similar in magnitude across age groups for self-harm thoughts.


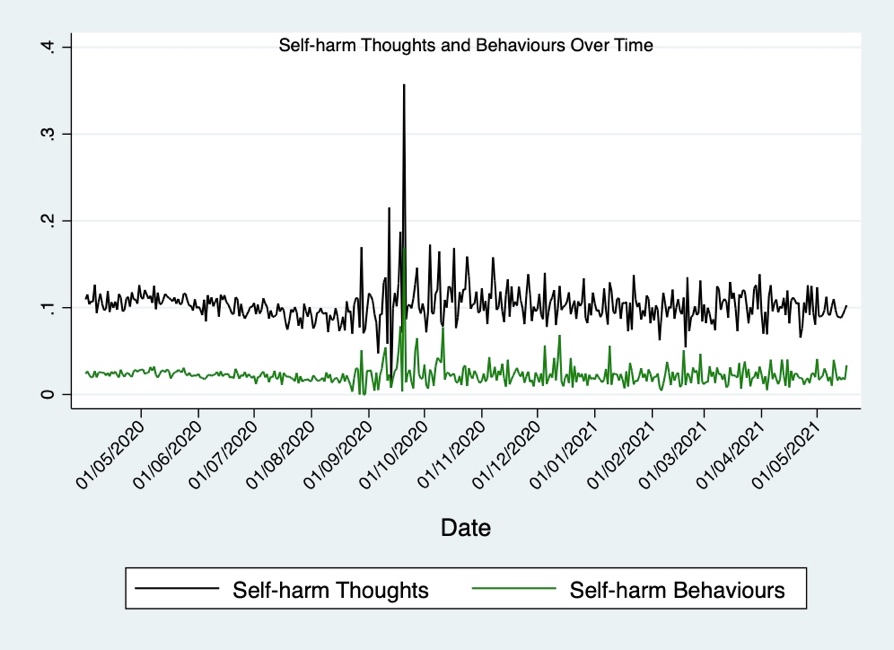


Figure S1a. Average proportion of the sample with self-harm thoughts and self-harm behaviours over time for the duration of the study period (1 April 2020 to 17 May 2021). Increased variability in data starting in August may be due to the change in data collection frequency from weekly to monthly that occurred at this time.


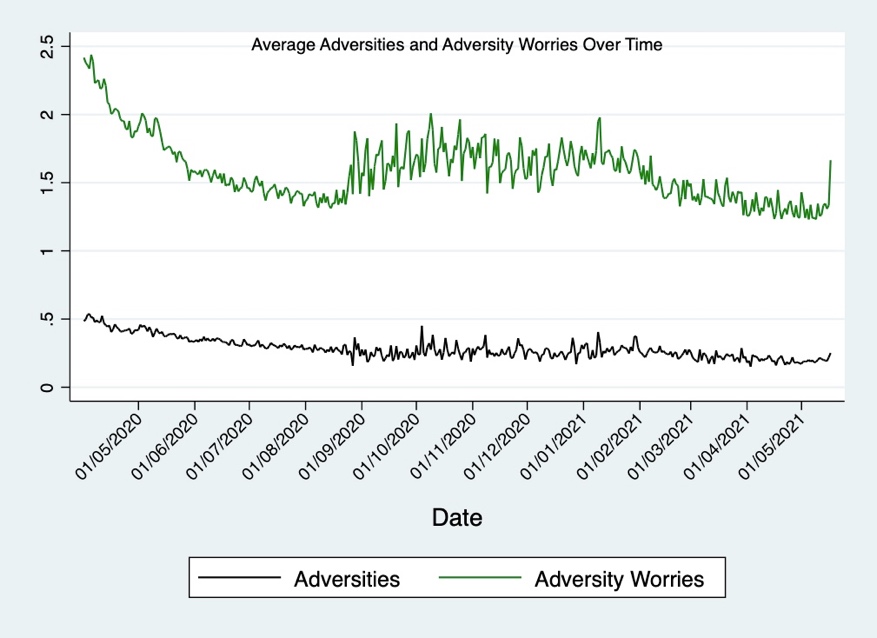


Figure S1b. Mean adversities and worries about adversity over time for the duration of the study period (1 April 2020 to 17 May 2021). Increased variability in data starting in August may be due to the change in data collection frequency from weekly to monthly that occurred at this time.


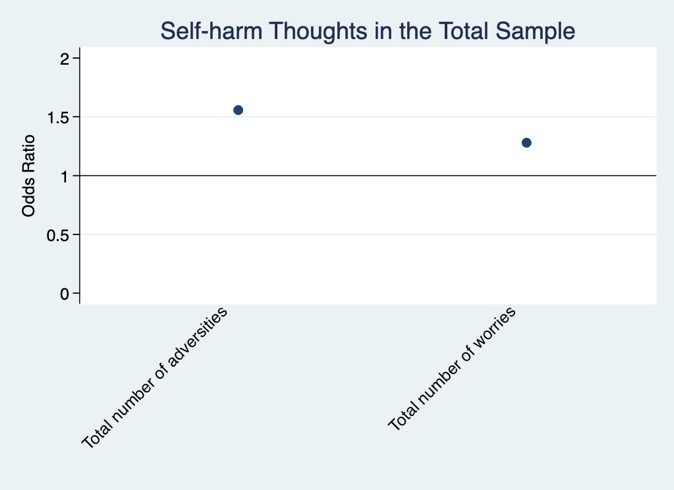

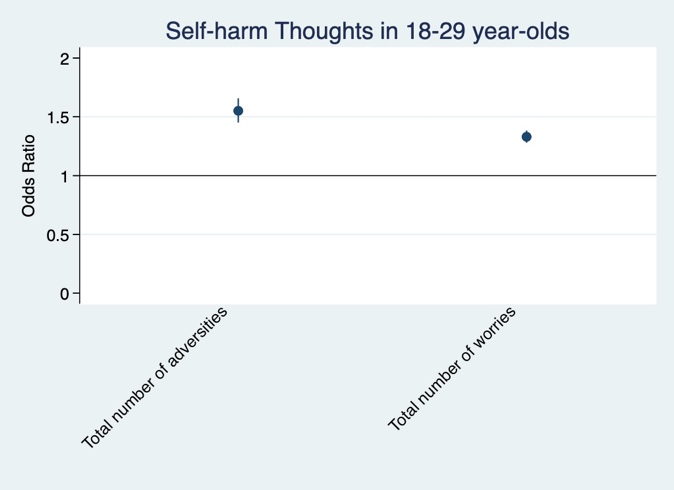

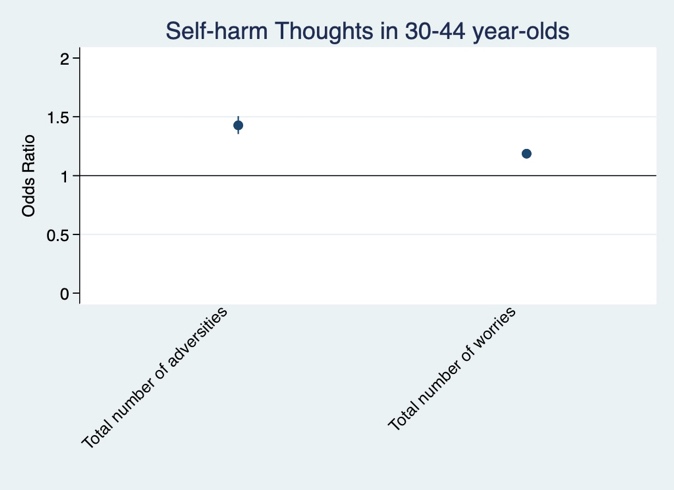

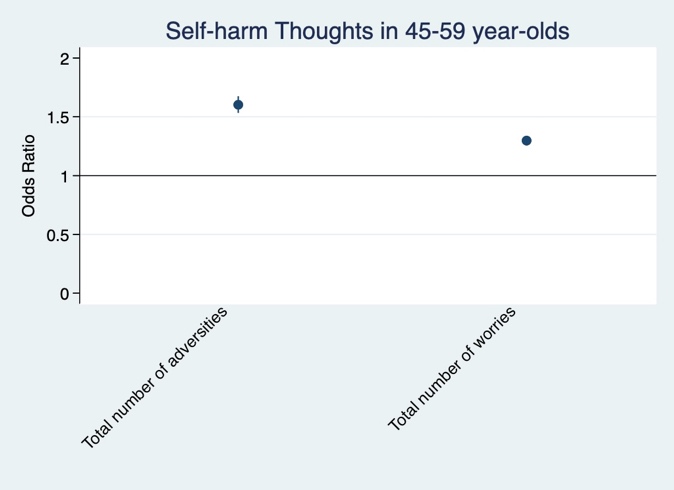

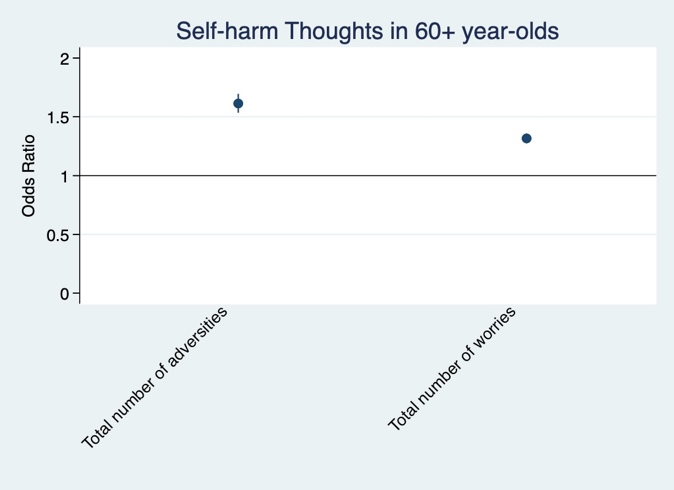


Figure S2. Associations between the total number adversity experiences and worries about adversity and change over time in self-harm thoughts derived from fixed effects models. Experiences and worries were entered simultaneously into the same model. Analyses were further adjusted for day of the week and time since lockdown began.


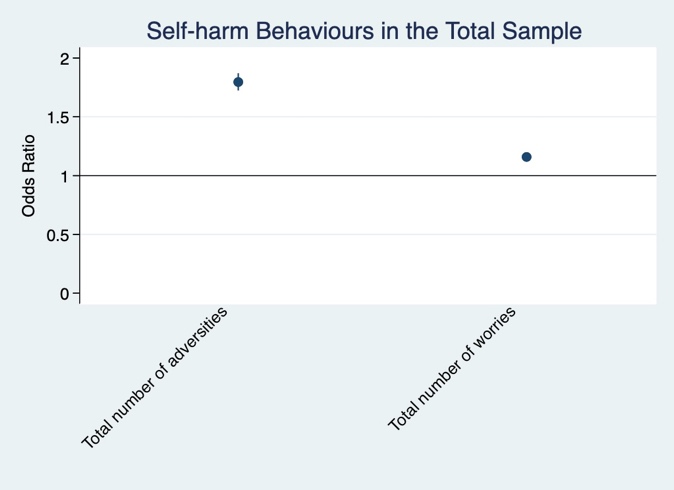

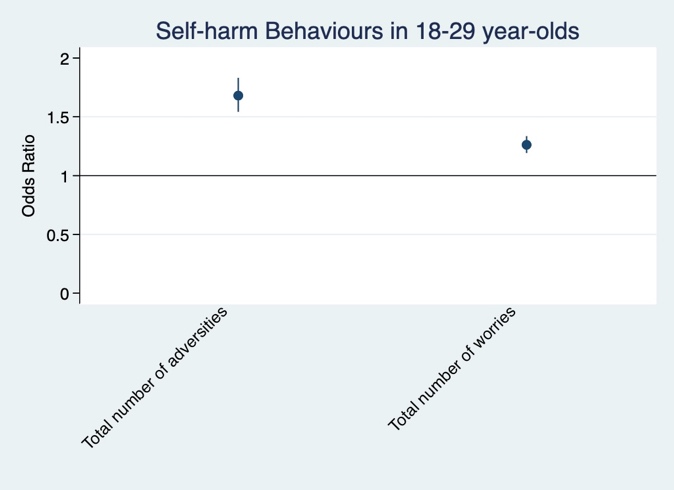

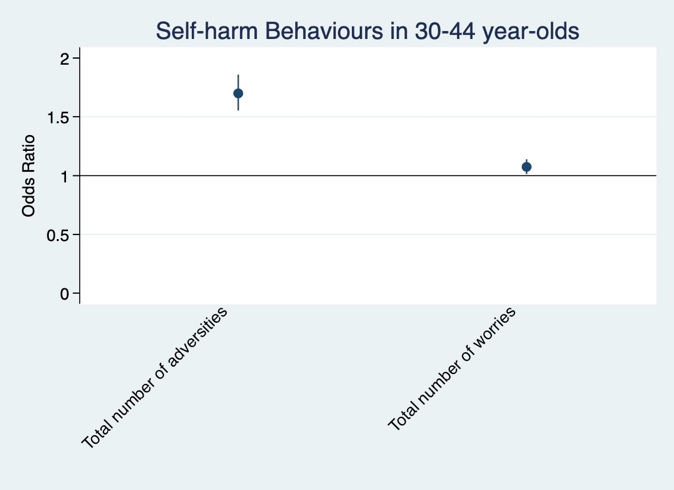

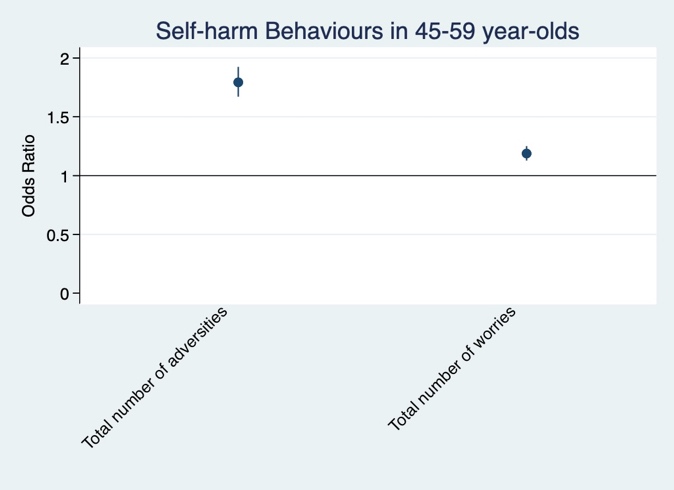

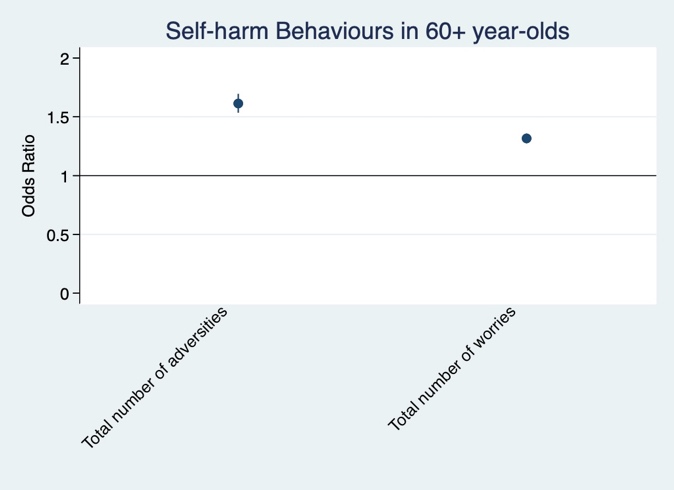


Figure S3. Associations between the total number adversity experiences and worries about adversity and change over time in self-harm behaviours derived from fixed effects models stratified by age. Experiences and worries were entered simultaneously into the same model. Analyses were further adjusted for day of the week and time since lockdown began.


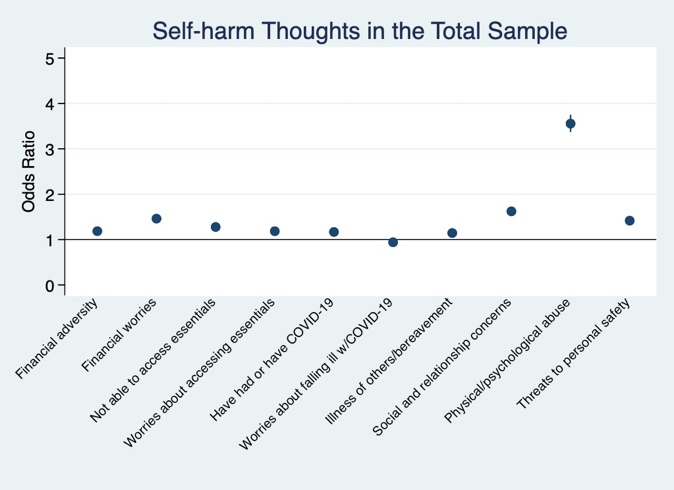

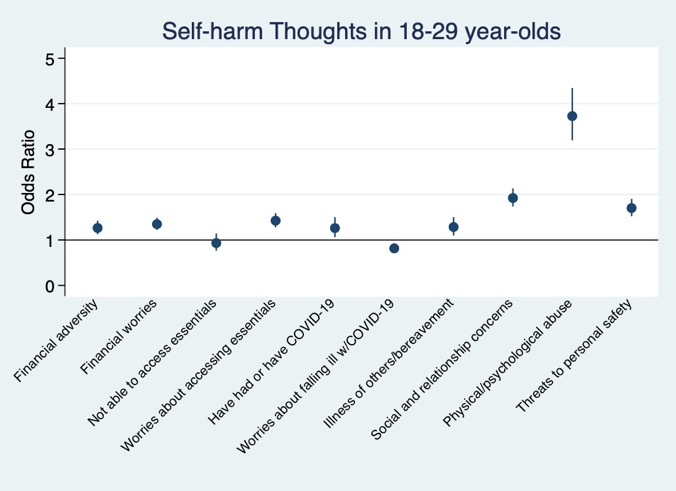

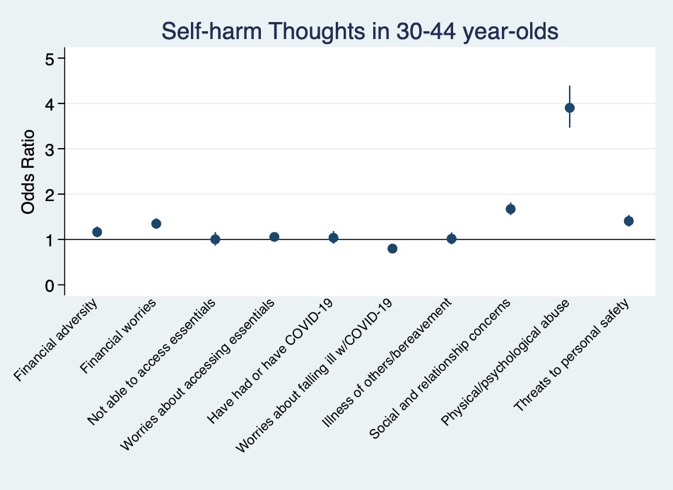

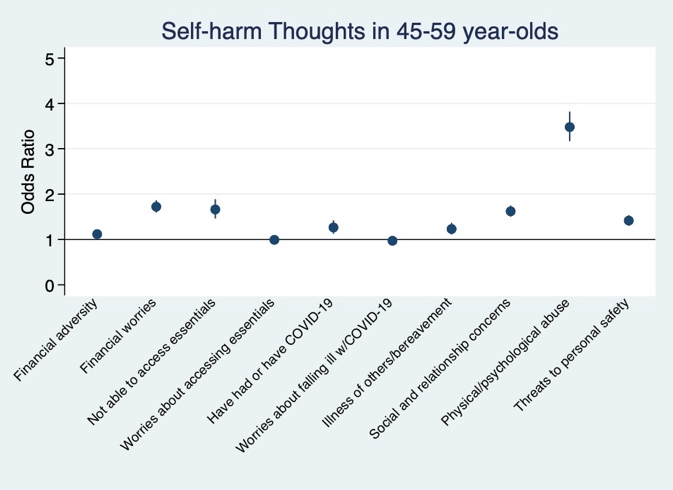

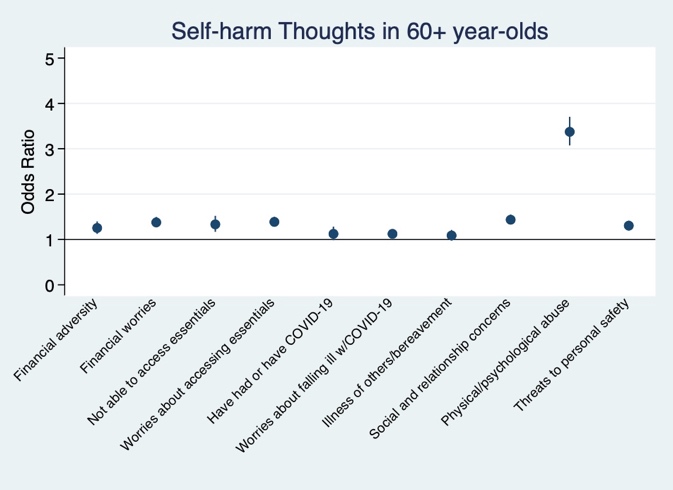


Figure S4. Associations between individual adversity experiences and worries about adversity and change over time in self-harm thoughts derived from fixed effects models. Experiences and worries were entered simultaneously into the same model. Analyses were further adjusted for day of the week and time since lockdown began.


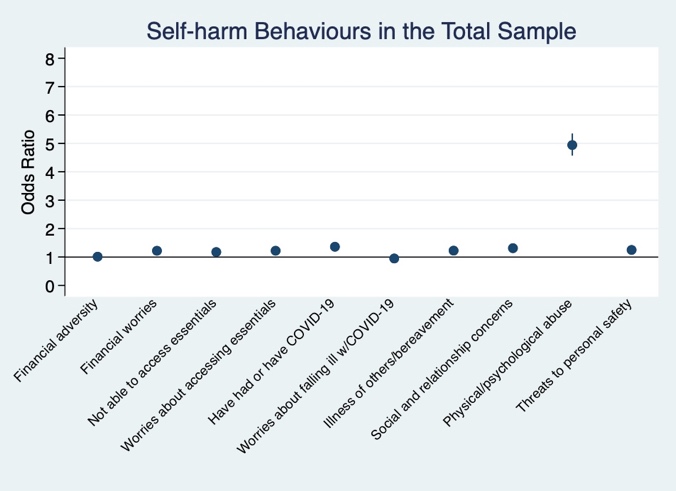

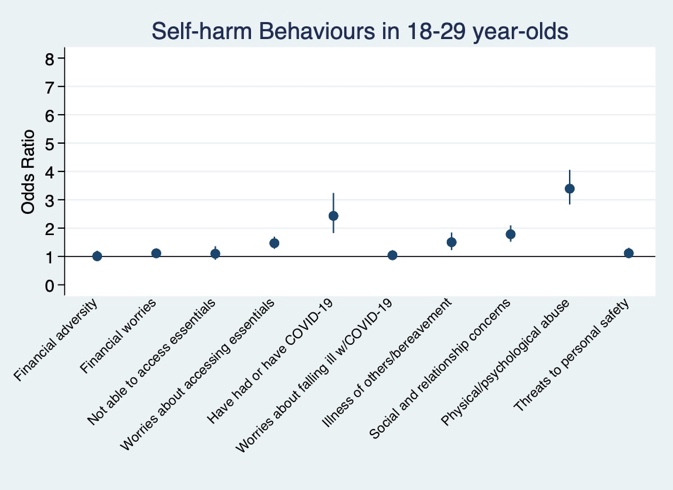

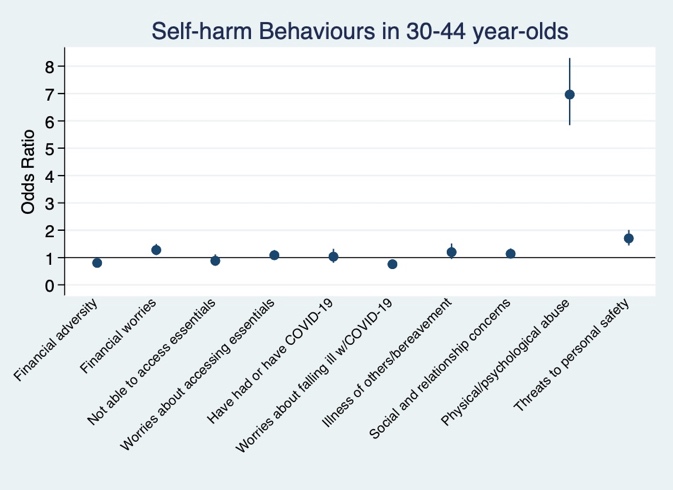

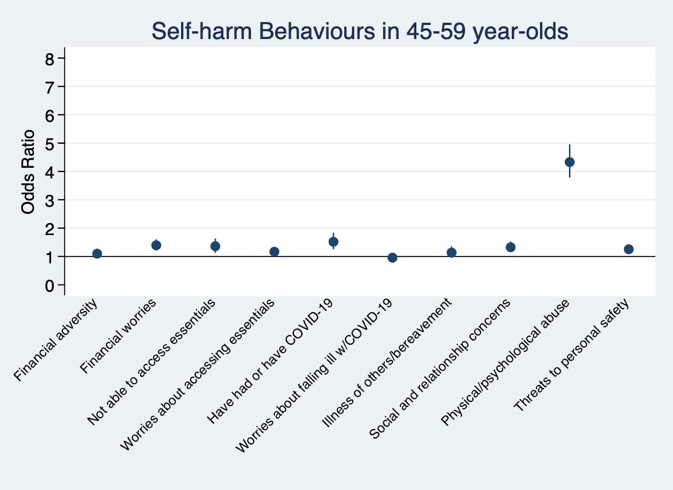

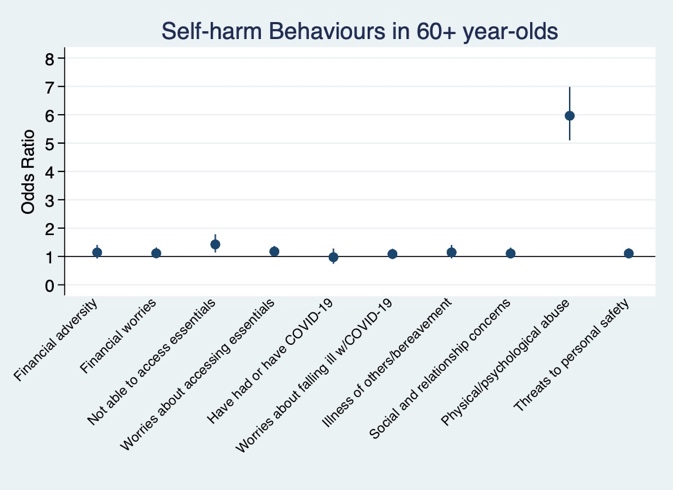


Figure S5. Associations between individual adversity experiences and worries about adversity and change over time in self-harm behaviours derived from fixed effects models. Experiences and worries were entered simultaneously into the same model. Analyses were further adjusted for day of the week and time since lockdown began.

Table S1. Characteristics of included and excluded participants, unweighted

|  | | Included | | Excluded | |
| --- | --- | --- | --- | --- | --- |
| Variable | | M | SD | M | SD |
| Female (ref male) | | 0.75 | 0.43 | 0.73 | 0.44 |
| Age |  |  |  |  |  |
|  | 18-29 | 0.07 | 0.26 | 0.17 | 0.37 |
|  | 30-44 | 0.25 | 0.43 | 0.36 | 0.48 |
|  | 45-59 | 0.35 | 0.48 | 0.29 | 0.46 |
|  | 60+ | 0.33 | 0.47 | 0.18 | 0.38 |
| Ethnic minority (ref white) | | 0.05 | 0.21 | 0.09 | 0.28 |
| Education |  |  |  |  |  |
| Up to GCSE | | 0.14 | 0.34 | 0.18 | 0.38 |
| A-levels or vocational | | 0.17 | 0.38 | 0.20 | 0.40 |
| Undergraduate | | 0.41 | 0.49 | 0.38 | 0.48 |
| Postgraduate | | 0.28 | 0.45 | 0.24 | 0.43 |
| Income |  |  |  |  |  |
| £90,000+ | | 0.11 | 0.31 | 0.11 | 0.31 |
| £60,000-90,000 | | 0.16 | 0.36 | 0.15 | 0.36 |
| £30,000-60,000 | | 0.35 | 0.48 | 0.33 | 0.47 |
| £16,000-30,000 | | 0.24 | 0.43 | 0.24 | 0.43 |
| < £16,000 | | 0.14 | 0.35 | 0.18 | 0.38 |
| Living arrangement | |  |  |  |  |
|  | Alone | 0.19 | 0.40 | 0.15 | 0.36 |
| With others, not including children | | 0.55 | 0.50 | 0.49 | 0.50 |
| With others, including children | | 0.26 | 0.44 | 0.36 | 0.48 |
| Keyworker (ref not) | | 0.23 | 0.42 | 0.29 | 0.45 |
| Long-term physical health condition | | 0.40 | 0.49 | 0.36 | 0.48 |
| Long-term mental health condition | | 0.18 | 0.39 | 0.23 | 0.42 |
| Number of individuals | | 49,324 | | 16,984 | |

Table S2. Socio-demographic characteristics for entire sample and for those with variation in self-harm thoughts and self-harm behaviours, unweighted and weighted

|  | | Whole sample | | | | Sample with variation in self-harm thoughts | | | | Sample with variation in self-harm behaviours | | | |
| --- | --- | --- | --- | --- | --- | --- | --- | --- | --- | --- | --- | --- | --- |
|  | | Unweighted | | Weighted | | Unweighted | | Weighted | | Unweighted | | Weighted | |
| Variable | | M | SD | M | SD | M | SD | M | SD | M | SD | M | SD |
| Female (ref male) | | 0.75 | 0.43 | 0.52 | 0.50 | 0.78 | 0.41 | 0.55 | 0.50 | 0.78 | 0.42 | 0.55 | 0.50 |
| Age |  |  |  |  |  |  |  |  |  |  |  |  |  |
|  | 18-29 | 0.07 | 0.26 | 0.13 | 0.33 | 0.10 | 0.30 | 0.18 | 0.38 | 0.14 | 0.35 | 0.23 | 0.42 |
|  | 30-44 | 0.25 | 0.43 | 0.21 | 0.40 | 0.29 | 0.46 | 0.25 | 0.43 | 0.32 | 0.47 | 0.25 | 0.43 |
|  | 45-59 | 0.35 | 0.48 | 0.29 | 0.45 | 0.35 | 0.48 | 0.29 | 0.46 | 0.35 | 0.48 | 0.30 | 0.46 |
|  | 60+ | 0.33 | 0.47 | 0.38 | 0.49 | 0.25 | 0.43 | 0.28 | 0.45 | 0.18 | 0.39 | 0.22 | 0.42 |
| Ethnic minority (ref white) | | 0.05 | 0.21 | 0.09 | 0.29 | 0.06 | 0.23 | 0.12 | 0.33 | 0.06 | 0.24 | 0.13 | 0.34 |
| Education |  |  |  |  |  |  |  |  |  |  |  |  |  |
| Up to GCSE | | 0.14 | 0.34 | 0.31 | 0.46 | 0.13 | 0.34 | 0.30 | 0.46 | 0.16 | 0.37 | 0.33 | 0.47 |
| A-levels or vocational | | 0.17 | 0.38 | 0.33 | 0.47 | 0.18 | 0.38 | 0.35 | 0.48 | 0.20 | 0.40 | 0.36 | 0.48 |
| Undergraduate | | 0.41 | 0.49 | 0.22 | 0.41 | 0.40 | 0.49 | 0.21 | 0.40 | 0.38 | 0.49 | 0.18 | 0.39 |
| Postgraduate | | 0.28 | 0.45 | 0.14 | 0.35 | 0.29 | 0.45 | 0.15 | 0.36 | 0.26 | 0.44 | 0.12 | 0.33 |
| Income |  |  |  |  |  |  |  |  |  |  |  |  |  |
| £90,000+ | | 0.11 | 0.31 | 0.08 | 0.27 | 0.09 | 0.28 | 0.06 | 0.25 | 0.07 | 0.25 | 0.05 | 0.22 |
| £60,000-90,000 | | 0.16 | 0.36 | 0.12 | 0.33 | 0.14 | 0.35 | 0.11 | 0.31 | 0.12 | 0.33 | 0.08 | 0.27 |
| £30,000-60,000 | | 0.35 | 0.48 | 0.33 | 0.47 | 0.34 | 0.47 | 0.30 | 0.46 | 0.30 | 0.46 | 0.25 | 0.44 |
| £16,000-30,000 | | 0.24 | 0.43 | 0.28 | 0.45 | 0.25 | 0.44 | 0.28 | 0.45 | 0.25 | 0.43 | 0.26 | 0.44 |
| < £16,000 | | 0.14 | 0.35 | 0.20 | 0.40 | 0.19 | 0.39 | 0.24 | 0.43 | 0.26 | 0.44 | 0.35 | 0.48 |
| Living arrangement | |  |  |  |  |  |  |  |  |  |  |  |  |
|  | Alone | 0.19 | 0.40 | 0.20 | 0.40 | 0.23 | 0.42 | 0.23 | 0.42 | 0.24 | 0.43 | 0.24 | 0.43 |
| With others, not including children | | 0.55 | 0.50 | 0.57 | 0.49 | 0.52 | 0.50 | 0.54 | 0.50 | 0.52 | 0.50 | 0.53 | 0.50 |
| With others, including children | | 0.26 | 0.44 | 0.23 | 0.42 | 0.25 | 0.43 | 0.23 | 0.42 | 0.24 | 0.43 | 0.23 | 0.42 |
| Keyworker (ref not) | | 0.23 | 0.42 | 0.21 | 0.40 | 0.23 | 0.42 | 0.20 | 0.40 | 0.23 | 0.42 | 0.19 | 0.39 |
| Long-term physical health condition | | 0.40 | 0.49 | 0.44 | 0.50 | 0.45 | 0.50 | 0.48 | 0.50 | 0.50 | 0.50 | 0.54 | 0.50 |
| Long-term mental health condition | | 0.18 | 0.39 | 0.19 | 0.39 | 0.34 | 0.47 | 0.34 | 0.48 | 0.52 | 0.50 | 0.52 | 0.50 |
| Number of individuals | | 49,324 | | | | 11,580 | | | | 3,747 | | | |

Note. Data in the weighted samples were weighted to the proportions of gender, age, ethnicity, country, and education obtained from the Office for National Statistics. Ethnic minority refers to Black, Asian and minority ethnicity. GCSE refers to General Certificate of Secondary Education.

Table S3. Descriptive statistics by number of times self-harm thoughts were reported in total sample (N = 49,324), weighted

|  | | Self-harm thoughts | | | | | |
| --- | --- | --- | --- | --- | --- | --- | --- |
|  | | Never  n = 36,455  (73.9%) | | Once or twice  n = 5,650  (11.5%) | | Thrice or more  n = 7,219  (14.6%) | |
| Variable | | M | SD | M | SD | M | SD |
| Gender (ref male) | Female | 0.51 | 0.50 | 0.55 | 0.50 | 0.53 | 0.50 |
| Age | 18-29 | 0.10 | 0.30 | 0.18 | 0.38 | 0.20 | 0.40 |
|  | 30-44 | 0.19 | 0.39 | 0.26 | 0.44 | 0.24 | 0.43 |
|  | 45-59 | 0.28 | 0.45 | 0.28 | 0.45 | 0.31 | 0.46 |
|  | 60+ | 0.43 | 0.49 | 0.27 | 0.44 | 0.25 | 0.43 |
| Ethnicity (ref white) | Ethnic minority | 0.08 | 0.28 | 0.13 | 0.33 | 0.12 | 0.32 |
| Education | Up to GCSE | 0.31 | 0.46 | 0.29 | 0.46 | 0.32 | 0.47 |
|  | A-levels or vocational | 0.32 | 0.47 | 0.35 | 0.48 | 0.36 | 0.48 |
|  | Undergraduate | 0.22 | 0.42 | 0.21 | 0.40 | 0.19 | 0.39 |
|  | Postgraduate | 0.15 | 0.35 | 0.15 | 0.36 | 0.13 | 0.34 |
| Income | £90,000+ | 0.09 | 0.27 | 0.08 | 0.26 | 0.05 | 0.22 |
|  | £60,000-90,000 | 0.13 | 0.33 | 0.12 | 0.33 | 0.08 | 0.27 |
|  | £30,000-60,000 | 0.34 | 0.47 | 0.30 | 0.46 | 0.28 | 0.45 |
|  | £16,000-30,000 | 0.28 | 0.45 | 0.28 | 0.45 | 0.28 | 0.45 |
|  | < £16,000 | 0.17 | 0.37 | 0.22 | 0.42 | 0.31 | 0.46 |
| Living arrangement | Alone | 0.18 | 0.38 | 0.20 | 0.40 | 0.26 | 0.44 |
| With others, not children | | 0.59 | 0.49 | 0.54 | 0.50 | 0.54 | 0.50 |
| With others, including children | | 0.23 | 0.42 | 0.26 | 0.44 | 0.21 | 0.40 |
| Keyworker (ref not) | Keyworker | 0.21 | 0.41 | 0.21 | 0.41 | 0.18 | 0.39 |
| Long-term physical health condition  Present | | 0.42 | 0.49 | 0.45 | 0.50 | 0.52 | 0.50 |
| Long-term mental health condition  Present | | 0.11 | 0.31 | 0.28 | 0.45 | 0.48 | 0.50 |

Note. Data were weighted to the proportions of gender, age, ethnicity, country, and education obtained from the Office for National Statistics. Ethnic minority refers to Black, Asian and minority ethnicity. GCSE refers to General Certificate of Secondary Education.

Table S4. Descriptive statistics by number of times self-harm behaviours were reported in total sample (N = 49,324), weighted

|  | | Self-harm behaviours | | | | | |
| --- | --- | --- | --- | --- | --- | --- | --- |
|  | | Never  n = 45,408  (92.1%) | | Once or twice  n = 2,424  (4.9%) | | Thrice or more  n = 1,492  (3.0%) | |
| Variable | | M | SD | M | SD | M | SD |
| Gender (ref male) | Female | 0.51 | 0.50 | 0.56 | 0.50 | 0.54 | 0.50 |
| Age | 18-29 | 0.11 | 0.32 | 0.23 | 0.42 | 0.27 | 0.44 |
|  | 30-44 | 0.20 | 0.40 | 0.26 | 0.44 | 0.23 | 0.42 |
|  | 45-59 | 0.29 | 0.45 | 0.29 | 0.45 | 0.32 | 0.47 |
|  | 60+ | 0.40 | 0.49 | 0.22 | 0.42 | 0.18 | 0.38 |
| Ethnicity (ref white) | Ethnic minority | 0.09 | 0.29 | 0.12 | 0.33 | 0.15 | 0.36 |
| Education | Up to GCSE | 0.31 | 0.46 | 0.34 | 0.48 | 0.34 | 0.47 |
|  | A-levels or vocational | 0.32 | 0.47 | 0.35 | 0.48 | 0.38 | 0.49 |
|  | Undergraduate | 0.22 | 0.41 | 0.18 | 0.38 | 0.18 | 0.38 |
|  | Postgraduate | 0.15 | 0.35 | 0.13 | 0.33 | 0.11 | 0.31 |
| Income | £90,000+ | 0.08 | 0.27 | 0.06 | 0.24 | 0.03 | 0.16 |
|  | £60,000-90,000 | 0.13 | 0.33 | 0.09 | 0.28 | 0.06 | 0.23 |
|  | £30,000-60,000 | 0.33 | 0.47 | 0.26 | 0.44 | 0.23 | 0.42 |
|  | £16,000-30,000 | 0.28 | 0.45 | 0.27 | 0.44 | 0.24 | 0.43 |
|  | < £16,000 | 0.18 | 0.38 | 0.32 | 0.47 | 0.45 | 0.50 |
| Living arrangement | Alone | 0.19 | 0.39 | 0.23 | 0.42 | 0.26 | 0.43 |
|  | With others, not including children | 0.58 | 0.49 | 0.53 | 0.50 | 0.54 | 0.50 |
|  | With others, including children | 0.23 | 0.42 | 0.25 | 0.43 | 0.20 | 0.40 |
| Keyworker (ref not) | Keyworker | 0.21 | 0.41 | 0.20 | 0.40 | 0.18 | 0.38 |
| Long-term physical health condition | Present | 0.43 | 0.50 | 0.53 | 0.50 | 0.56 | 0.50 |
| Long-term mental health condition | Present | 0.15 | 0.36 | 0.47 | 0.50 | 0.65 | 0.48 |

Note. Data were weighted to the proportions of gender, age, ethnicity, country, and education obtained from the Office for National Statistics. Ethnic minority refers to Black, Asian and minority ethnicity. GCSE refers to General Certificate of Secondary Education.

Table S5. Descriptive statistics for predictor and outcome variables amongst individuals with variation in each outcome variable

|  |  | Sample with variation in self-harm thoughts | | | | Sample with variation in self-harm behaviours | | | |
| --- | --- | --- | --- | --- | --- | --- | --- | --- | --- |
| Variable |  | Overall Mean | Overall SD | Between SD | Within SD | Overall Mean | Overall SD | Between SD | Within SD |
| Outcomes | Self-harm thoughts | 0.28 | 0.45 | 0.26 | 0.36 | 0.47 | 0.50 | 0.36 | 0.34 |
|  | Self-harm behaviours | 0.04 | 0.20 | 0.14 | 0.16 | 0.20 | 0.40 | 0.23 | 0.33 |
| Adversity experiences | |  |  |  |  |  |  |  |  |
|  | Total adversities (0-5) | 0.48 | 0.71 | 0.57 | 0.47 | 0.60 | 0.80 | 0.66 | 0.52 |
|  | Financial | 0.15 | 0.36 | 0.29 | 0.24 | 0.17 | 0.38 | 0.30 | 0.25 |
|  | Accessing essentials | 0.04 | 0.20 | 0.15 | 0.16 | 0.07 | 0.26 | 0.19 | 0.20 |
|  | COVID-19 illness | 0.12 | 0.32 | 0.27 | 0.19 | 0.12 | 0.33 | 0.27 | 0.20 |
| Illness of others/bereavement | | 0.06 | 0.24 | 0.12 | 0.21 | 0.07 | 0.26 | 0.15 | 0.22 |
| Physical/psychological abuse | | 0.11 | 0.31 | 0.23 | 0.22 | 0.17 | 0.38 | 0.28 | 0.26 |
| Worries |  |  |  |  |  |  |  |  |  |
|  | Total worries (0-5) | 2.18 | 1.21 | 0.96 | 0.78 | 2.36 | 1.28 | 1.01 | 0.81 |
|  | Financial | 0.60 | 0.49 | 0.37 | 0.31 | 0.64 | 0.48 | 0.36 | 0.31 |
|  | Accessing essentials | 0.19 | 0.39 | 0.27 | 0.30 | 0.25 | 0.43 | 0.30 | 0.33 |
|  | COVID-19 illness | 0.58 | 0.50 | 0.38 | 0.32 | 0.48 | 0.50 | 0.38 | 0.32 |
| Social/relationship concerns | | 0.77 | 0.42 | 0.27 | 0.32 | 0.79 | 0.41 | 0.27 | 0.31 |
|  | Threats to safety | 0.14 | 0.35 | 0.24 | 0.27 | 0.20 | 0.40 | 0.28 | 0.30 |
| Number of observations | | 206,714 | | | | 63,767 | | | |
|  | Number of individuals | 11,580 | | | | 3,747 | | | |

Table S6. Fixed-effects logistic regression models predicting within-individual change in self-harm thoughts from individual categories of adversity experiences and worries accounting for anxiety symptoms

|  | Self-harm thoughts | | | | | | | | | | | | | | |
| --- | --- | --- | --- | --- | --- | --- | --- | --- | --- | --- | --- | --- | --- | --- | --- |
|  | Total sample | | | Ages 18-29 | | | Ages 30-44 | | | Ages 45-59 | | | Ages 60+ | | |
| Variable | OR | 95% CI  lower | 95% CI  upper | OR | 95% CI  lower | 95% CI  upper | OR | 95% CI  lower | 95% CI  upper | OR | 95% CI  lower | 95% CI  upper | OR | 95% CI  lower | 95% CI  upper |
| Anxiety symptoms | 1.34 | 1.33 | 1.34 | 1.32 | 1.30 | 1.33 | 1.32 | 1.30 | 1.33 | 1.34 | 1.33 | 1.35 | 1.36 | 1.35 | 1.38 |
| Adversity experiences |  |  |  |  |  |  |  |  |  |  |  |  |  |  |  |
| Financial | 1.01 | 0.96 | 1.07 | 1.06 | 0.93 | 1.20 | 1.04 | 0.93 | 1.16 | 0.97 | 0.88 | 1.06 | 1.03 | 0.92 | 1.16 |
| Accessing essentials | 1.17 | 1.09 | 1.27 | 0.84 | 0.67 | 1.04 | 0.91 | 0.78 | 1.07 | 1.49 | 1.30 | 1.71 | 1.27 | 1.11 | 1.47 |
| COVID-19 illness | 1.11 | 1.03 | 1.20 | 1.19 | 0.99 | 1.44 | 1.00 | 0.86 | 1.15 | 1.23 | 1.09 | 1.40 | 1.05 | 0.91 | 1.22 |
| Illness of others/ bereavement | 0.97 | 0.91 | 1.04 | 1.07 | 0.90 | 1.26 | 0.92 | 0.80 | 1.06 | 1.06 | 0.95 | 1.19 | 0.89 | 0.79 | 1.00 |
| Physical/psychological abuse | 2.68 | 2.52 | 2.83 | 3.28 | 2.78 | 3.87 | 3.01 | 2.65 | 3.42 | 2.59 | 2.35 | 2.87 | 2.36 | 2.13 | 2.61 |
| Worries |  |  |  |  |  |  |  |  |  |  |  |  |  |  |  |
| Financial | 1.16 | 1.11 | 1.22 | 1.11 | 1.00 | 1.23 | 1.05 | 0.96 | 1.16 | 1.30 | 1.19 | 1.41 | 1.18 | 1.08 | 1.29 |
| Accessing essentials | 1.02 | 0.97 | 1.07 | 1.20 | 1.06 | 1.35 | 0.92 | 0.84 | 1.01 | 0.86 | 0.79 | 0.94 | 1.18 | 1.09 | 1.29 |
| COVID-19 illness | 0.84 | 0.80 | 0.88 | 0.75 | 0.67 | 0.83 | 0.74 | 0.68 | 0.81 | 0.85 | 0.78 | 0.92 | 0.98 | 0.90 | 1.07 |
| Social/relationship concerns | 1.31 | 1.26 | 1.38 | 1.53 | 1.36 | 1.71 | 1.34 | 1.22 | 1.47 | 1.30 | 1.20 | 1.41 | 1.22 | 1.12 | 1.33 |
| Threats to safety | 1.12 | 1.06 | 1.17 | 1.39 | 1.23 | 1.57 | 1.07 | 0.97 | 1.18 | 1.12 | 1.03 | 1.22 | 1.05 | 0.96 | 1.15 |
| Number of observations | 206,005 | | | 16,434 | | | 53,586 | | | 74,421 | | | 61,564 | | |
| Number of individuals | 11,548 | | | 1,164 | | | 3,387 | | | 4,060 | | | 2,937 | | |

Note. Adversity experiences and worries variables are weighted to the proportions of gender, age, ethnicity, country, and education obtained from the Office for National Statistics. Individual adversity experiences and worries variables are binary. Analyses were further adjusted for day of the week and time since lockdown began. OR = odds ratio. CI = confidence interval.

Table S7. Fixed-effects logistic regression models predicting within-individual change in self-harm behaviours from individual categories of adversity experiences and worries accounting for anxiety symptoms

|  |  | Self-harm behaviours | | | | | | | | | | | | | | |
| --- | --- | --- | --- | --- | --- | --- | --- | --- | --- | --- | --- | --- | --- | --- | --- | --- |
|  |  | Total sample | | | Ages 18-29 | | | Ages 30-44 | | | Ages 45-59 | | | Ages 60+ | | |
| Variable |  | OR | 95% CI  lower | 95% CI  upper | OR | 95% CI  lower | 95% CI  upper | OR | 95% CI  lower | 95% CI  upper | OR | 95% CI  lower | 95% CI  upper | OR | 95% CI  lower | 95% CI  upper |
| Anxiety symptoms | | 1.18 | 1.17 | 1.19 | 1.16 | 1.14 | 1.18 | 1.19 | 1.17 | 1.21 | 1.19 | 1.17 | 1.21 | 1.21 | 1.19 | 1.23 |
| Adversity experiences | |  |  |  |  |  |  |  |  |  |  |  |  |  |  |  |
|  | Financial | 0.95 | 0.87 | 1.04 | 1.03 | 0.86 | 1.24 | 0.76 | 0.62 | 0.92 | 1.03 | 0.88 | 1.20 | 0.90 | 0.73 | 1.12 |
| Accessing essentials | | 1.15 | 1.03 | 1.28 | 1.04 | 0.84 | 1.30 | 0.93 | 0.73 | 1.18 | 1.26 | 1.05 | 1.52 | 1.47 | 1.17 | 1.84 |
| COVID-19 illness | | 1.35 | 1.20 | 1.53 | 2.77 | 2.07 | 3.72 | 1.00 | 0.78 | 1.29 | 1.46 | 1.20 | 1.78 | 0.92 | 0.69 | 1.22 |
| Illness of others/bereavement | | 1.11 | 1.01 | 1.24 | 1.39 | 1.12 | 1.71 | 1.10 | 0.86 | 1.40 | 1.06 | 0.89 | 1.27 | 1.00 | 0.81 | 1.24 |
| Physical/ psychological abuse | | 4.14 | 3.82 | 4.49 | 3.09 | 2.58 | 3.71 | 6.33 | 5.28 | 7.60 | 3.54 | 3.08 | 4.07 | 4.54 | 3.85 | 5.35 |
| Worries | |  |  |  |  |  |  |  |  |  |  |  |  |  |  |  |
|  | Financial | 1.08 | 1.00 | 1.17 | 0.96 | 0.82 | 1.12 | 1.09 | 0.92 | 1.29 | 1.24 | 1.07 | 1.43 | 1.05 | 0.88 | 1.25 |
| Accessing essentials | | 1.17 | 1.08 | 1.26 | 1.41 | 1.22 | 1.64 | 1.02 | 0.87 | 1.19 | 1.11 | 0.97 | 1.28 | 1.12 | 0.96 | 1.31 |
| COVID-19 illness | | 0.91 | 0.84 | 0.98 | 1.00 | 0.85 | 1.18 | 0.73 | 0.62 | 0.86 | 0.93 | 0.81 | 1.07 | 1.00 | 0.85 | 1.19 |
| Social/relationship concerns | | 1.13 | 1.04 | 1.22 | 1.52 | 1.29 | 1.79 | 0.97 | 0.82 | 1.14 | 1.16 | 1.00 | 1.34 | 0.96 | 0.80 | 1.15 |
| Threats to safety | | 1.07 | 0.99 | 1.15 | 0.98 | 0.83 | 1.15 | 1.47 | 1.24 | 1.74 | 1.03 | 0.90 | 1.18 | 0.96 | 0.82 | 1.13 |
| Number of observations | | 63,383 | | | 7,654 | | | 18,540 | | | 23,707 | | | 13,482 | | |
| Number of individuals | | 3,728 | | | 527 | | | 1,197 | | | 1,322 | | | 682 | | |

Note. Adversity experiences and worries variables are weighted to the proportions of gender, age, ethnicity, country, and education obtained from the Office for National Statistics. Individual adversity experiences and worries variables are binary. Analyses were further adjusted for day of the week and time since lockdown began. OR = odds ratio. CI = confidence interval.

Table S8. Fixed-effects logistic regression models predicting within-individual change in self-harm thoughts from individual categories of adversity experiences and worries accounting for depressive symptoms

|  | Self-harm thoughts | | | | | | | | | | | | | | | |
| --- | --- | --- | --- | --- | --- | --- | --- | --- | --- | --- | --- | --- | --- | --- | --- | --- |
|  | Total sample | | | Ages 18-29 | | | Ages 30-44 | | | Ages 45-59 | | | Ages 60+ | | | |
| Variable | OR | 95% CI  lower | 95% CI  upper | OR | 95% CI  lower | 95% CI  upper | OR | 95% CI  lower | 95% CI  upper | OR | 95% CI  lower | 95% CI  upper | OR | 95% CI  lower | 95% CI  upper |  |
| Depressive symptoms | 1.37 | 1.37 | 1.38 | 1.35 | 1.33 | 1.36 | 1.37 | 1.36 | 1.38 | 1.38 | 1.37 | 1.39 | 1.39 | 1.38 | 1.41 |  |
| Adversity experiences |  |  |  |  |  |  |  |  |  |  |  |  |  |  |  |  |
| Financial | 1.00 | 0.94 | 1.05 | 1.06 | 0.93 | 1.21 | 0.97 | 0.87 | 1.09 | 0.96 | 0.87 | 1.05 | 1.06 | 0.94 | 1.20 |  |
| Accessing essentials | 1.13 | 1.04 | 1.22 | 0.77 | 0.61 | 0.96 | 0.84 | 0.71 | 0.99 | 1.51 | 1.31 | 1.74 | 1.22 | 1.05 | 1.41 |  |
| COVID-19 illness | 1.05 | 0.97 | 1.13 | 1.29 | 1.06 | 1.57 | 0.91 | 0.78 | 1.06 | 1.14 | 1.00 | 1.30 | 0.97 | 0.83 | 1.12 |  |
| Illness of others/ bereavement | 0.97 | 0.90 | 1.03 | 1.01 | 0.85 | 1.20 | 0.88 | 0.76 | 1.02 | 1.05 | 0.93 | 1.17 | 0.94 | 0.84 | 1.06 |  |
| Physical/ psychological abuse | 2.65 | 2.50 | 2.81 | 3.07 | 2.60 | 3.63 | 2.77 | 2.43 | 3.16 | 2.59 | 2.34 | 2.88 | 2.50 | 2.26 | 2.78 |  |
| Worries |  |  |  |  |  |  |  |  |  |  |  |  |  |  |  |  |
| Financial | 1.18 | 1.13 | 1.24 | 1.15 | 1.03 | 1.27 | 1.05 | 0.96 | 1.15 | 1.30 | 1.19 | 1.42 | 1.20 | 1.09 | 1.31 |  |
| Accessing essentials | 1.01 | 0.96 | 1.05 | 1.23 | 1.09 | 1.38 | 0.90 | 0.82 | 0.99 | 0.84 | 0.77 | 0.92 | 1.16 | 1.06 | 1.26 |  |
| COVID-19 illness | 0.90 | 0.86 | 0.94 | 0.83 | 0.75 | 0.93 | 0.80 | 0.73 | 0.87 | 0.90 | 0.83 | 0.98 | 1.01 | 0.92 | 1.10 |  |
| Social/ relationship concerns | 1.32 | 1.26 | 1.38 | 1.58 | 1.41 | 1.77 | 1.31 | 1.19 | 1.43 | 1.32 | 1.21 | 1.44 | 1.23 | 1.13 | 1.34 |  |
| Threats to safety | 1.16 | 1.11 | 1.22 | 1.47 | 1.30 | 1.67 | 1.10 | 1.00 | 1.22 | 1.16 | 1.06 | 1.27 | 1.09 | 1.00 | 1.19 |  |
| Number of observations | 206,714 | | | 16,495 | | | 53,752 | | | 74,635 | | | 61,832 | | | |
| Number of individuals | 11,580 | | | 1,168 | | | 3,394 | | | 4,069 | | | 2,949 | | | |

Note. Adversity experiences and worries variables are weighted to the proportions of gender, age, ethnicity, country, and education obtained from the Office for National Statistics. Individual adversity experiences and worries variables are binary. Analyses were further adjusted for day of the week and time since lockdown began. OR = odds ratio. CI = confidence interval.

Table S9. Fixed-effects logistic regression models predicting within-individual change in self-harm behaviours from individual categories of adversity experiences and worries accounting for depressive symptoms

|  | Self-harm behaviours | | | | | | | | | | | | | | |
| --- | --- | --- | --- | --- | --- | --- | --- | --- | --- | --- | --- | --- | --- | --- | --- |
|  | Total sample | | | Ages 18-29 | | | Ages 30-44 | | | Ages 45-59 | | | Ages 60+ | | |
| Variable | OR | 95% CI  lower | 95% CI  upper | OR | 95% CI  lower | 95% CI  upper | OR | 95% CI  lower | 95% CI  upper | OR | 95% CI  lower | 95% CI  upper | OR | 95% CI  lower | 95% CI  upper |
| Depressive symptoms | 1.20 | 1.19 | 1.21 | 1.18 | 1.16 | 1.20 | 1.21 | 1.19 | 1.23 | 1.20 | 1.19 | 1.22 | 1.21 | 1.19 | 1.23 |
| Adversity experiences |  |  |  |  |  |  |  |  |  |  |  |  |  |  |  |
| Financial | 0.92 | 0.83 | 1.00 | 0.96 | 0.80 | 1.16 | 0.76 | 0.62 | 0.93 | 0.97 | 0.83 | 1.12 | 0.95 | 0.76 | 1.18 |
| Accessing essentials | 1.09 | 0.97 | 1.21 | 0.97 | 0.78 | 1.21 | 0.85 | 0.67 | 1.09 | 1.22 | 1.01 | 1.48 | 1.39 | 1.10 | 1.75 |
| COVID-19 illness | 1.32 | 1.17 | 1.50 | 2.99 | 2.20 | 4.05 | 0.88 | 0.68 | 1.14 | 1.49 | 1.22 | 1.82 | 0.91 | 0.68 | 1.20 |
| Illness of others/bereavement | 1.12 | 1.01 | 1.24 | 1.44 | 1.17 | 1.77 | 1.09 | 0.85 | 1.39 | 1.03 | 0.87 | 1.23 | 1.06 | 0.85 | 1.31 |
| Physical/ psychological abuse | 3.92 | 3.62 | 4.26 | 2.96 | 2.47 | 3.55 | 5.84 | 4.86 | 7.02 | 3.41 | 2.96 | 3.90 | 4.30 | 3.65 | 5.07 |
| Worries |  |  |  |  |  |  |  |  |  |  |  |  |  |  |  |
| Financial | 1.09 | 1.00 | 1.18 | 1.01 | 0.87 | 1.17 | 1.11 | 0.94 | 1.31 | 1.23 | 1.07 | 1.42 | 1.01 | 0.85 | 1.21 |
| Accessing essentials | 1.14 | 1.05 | 1.22 | 1.40 | 1.20 | 1.63 | 0.99 | 0.84 | 1.16 | 1.10 | 0.96 | 1.26 | 1.05 | 0.90 | 1.23 |
| COVID-19 illness | 0.95 | 0.88 | 1.03 | 1.07 | 0.91 | 1.26 | 0.76 | 0.65 | 0.90 | 0.95 | 0.83 | 1.09 | 1.06 | 0.90 | 1.26 |
| Social/ relationship concerns | 1.14 | 1.05 | 1.24 | 1.57 | 1.33 | 1.86 | 0.95 | 0.80 | 1.12 | 1.18 | 1.02 | 1.36 | 0.94 | 0.78 | 1.13 |
| Threats to safety | 1.09 | 1.01 | 1.18 | 1.01 | 0.85 | 1.19 | 1.49 | 1.26 | 1.77 | 1.08 | 0.94 | 1.23 | 0.95 | 0.81 | 1.12 |
| Number of observations | 63,767 | | | 7,687 | | | 18,594 | | | 23,859 | | | 13,627 | | |
| Number of individuals | 3,747 | | | 530 | | | 1,201 | | | 1,329 | | | 687 | | |

Note. Adversity experiences and worries variables are weighted to the proportions of gender, age, ethnicity, country, and education obtained from the Office for National Statistics. Individual adversity experiences and worries variables are binary. Analyses were further adjusted for day of the week and time since lockdown began. OR = odds ratio. CI = confidence interval.

Table S10. Fixed-effects logistic regression models predicting within-individual change in self-harm thoughts from individual categories of adversity experiences and worries with physical abuse and psychological abuse as individual adversity experiences

|  |  | Self-harm thoughts | | | | | | | | | | | | | | |
| --- | --- | --- | --- | --- | --- | --- | --- | --- | --- | --- | --- | --- | --- | --- | --- | --- |
|  |  | Total sample | | | Ages 18-29 | | | Ages 30-44 | | | Ages 45-59 | | | Ages 60+ | | |
| Variable |  | OR | 95% CI  lower | 95% CI  upper | OR | 95% CI  lower | 95% CI  upper | OR | 95% CI  lower | 95% CI  upper | OR | 95% CI  lower | 95% CI  upper | OR | 95% CI  lower | 95% CI  upper |
| Adversity experiences | |  |  |  |  |  |  |  |  |  |  |  |  |  |  |  |
|  | Financial | 1.18 | 1.13 | 1.24 | 1.26 | 1.12 | 1.42 | 1.16 | 1.04 | 1.28 | 1.12 | 1.03 | 1.22 | 1.25 | 1.12 | 1.40 |
|  | Accessing essentials | 1.28 | 1.19 | 1.38 | 0.94 | 0.77 | 1.15 | 1.01 | 0.87 | 1.17 | 1.66 | 1.46 | 1.89 | 1.34 | 1.17 | 1.53 |
|  | COVID-19 illness | 1.16 | 1.09 | 1.25 | 1.25 | 1.05 | 1.49 | 1.04 | 0.90 | 1.19 | 1.25 | 1.12 | 1.41 | 1.14 | 0.99 | 1.30 |
| Illness of others/bereavement | | 1.15 | 1.08 | 1.22 | 1.29 | 1.11 | 1.51 | 1.02 | 0.89 | 1.16 | 1.23 | 1.11 | 1.37 | 1.09 | 0.98 | 1.21 |
|  | Physical abuse | 2.86 | 2.58 | 3.17 | 4.72 | 3.18 | 7.01 | 4.97 | 3.87 | 6.38 | 2.60 | 2.15 | 3.13 | 2.30 | 1.97 | 2.68 |
|  | Psychological abuse | 3.05 | 2.88 | 3.23 | 3.25 | 2.77 | 3.81 | 3.05 | 2.69 | 3.47 | 3.07 | 2.78 | 3.39 | 2.93 | 2.65 | 3.24 |
| Worries |  |  |  |  |  |  |  |  |  |  |  |  |  |  |  |  |
|  | Financial | 1.46 | 1.40 | 1.52 | 1.33 | 1.21 | 1.47 | 1.36 | 1.25 | 1.48 | 1.73 | 1.60 | 1.87 | 1.38 | 1.27 | 1.50 |
|  | Accessing essentials | 1.18 | 1.13 | 1.23 | 1.42 | 1.27 | 1.58 | 1.06 | 0.97 | 1.16 | 0.99 | 0.92 | 1.07 | 1.38 | 1.28 | 1.49 |
|  | COVID-19 illness | 0.94 | 0.91 | 0.98 | 0.82 | 0.75 | 0.91 | 0.80 | 0.74 | 0.87 | 0.97 | 0.90 | 1.05 | 1.13 | 1.05 | 1.22 |
| Social/relationship concerns | | 1.63 | 1.56 | 1.69 | 1.92 | 1.73 | 2.14 | 1.69 | 1.55 | 1.83 | 1.62 | 1.51 | 1.75 | 1.44 | 1.33 | 1.56 |
|  | Threats to safety | 1.40 | 1.34 | 1.46 | 1.70 | 1.52 | 1.90 | 1.41 | 1.29 | 1.55 | 1.40 | 1.29 | 1.52 | 1.28 | 1.18 | 1.39 |
| Number of observations | | 206,714 | | | 16,495 | | | 53,752 | | | 74,635 | | | 61,832 | | |
|  | Number of individuals | 11,580 | | | 1,168 | | | 3,394 | | | 4,069 | | | 2,949 | | |

Note. Adversity experiences and worries variables are weighted to the proportions of gender, age, ethnicity, country, and education obtained from the Office for National Statistics. Individual adversity experiences and worries variables are binary. Analyses were further adjusted for day of the week and time since lockdown began. OR = odds ratio. CI = confidence interval.

Table S11. Fixed-effects logistic regression models predicting within-individual change in self-harm behaviours from individual categories of adversity experiences and worries with physical abuse and psychological abuse as individual adversity experiences

|  |  | Self-harm behaviours | | | | | | | | | | | | | | |
| --- | --- | --- | --- | --- | --- | --- | --- | --- | --- | --- | --- | --- | --- | --- | --- | --- |
|  |  | Total sample | | | Ages 18-29 | | | Ages 30-44 | | | Ages 45-59 | | | Ages 60+ | | |
| Variable |  | OR | 95% CI  lower | 95% CI  upper | OR | 95% CI  lower | 95% CI  upper | OR | 95% CI  lower | 95% CI  upper | OR | 95% CI  lower | 95% CI  upper | OR | 95% CI  lower | 95% CI  upper |
| Adversity experiences | |  |  |  |  |  |  |  |  |  |  |  |  |  |  |  |
|  | Financial | 1.01 | 0.92 | 1.11 | 0.96 | 0.80 | 1.16 | 0.78 | 0.64 | 0.95 | 1.13 | 0.97 | 1.31 | 1.11 | 0.89 | 1.37 |
|  | Accessing essentials | 1.19 | 1.07 | 1.33 | 1.09 | 0.87 | 1.37 | 0.91 | 0.72 | 1.16 | 1.36 | 1.13 | 1.64 | 1.49 | 1.18 | 1.89 |
|  | COVID-19 illness | 1.33 | 1.17 | 1.50 | 2.33 | 1.74 | 3.12 | 1.00 | 0.78 | 1.30 | 1.44 | 1.19 | 1.75 | 1.01 | 0.76 | 1.33 |
| Illness of others/bereavement | | 1.22 | 1.10 | 1.35 | 1.54 | 1.24 | 1.91 | 1.21 | 0.95 | 1.54 | 1.11 | 0.93 | 1.33 | 1.14 | 0.92 | 1.40 |
|  | Physical abuse | 8.85 | 7.85 | 9.96 | 19.53 | 13.53 | 28.19 | 15.70 | 11.62 | 21.21 | 5.77 | 4.74 | 7.02 | 7.83 | 6.36 | 9.63 |
|  | Psychological abuse | 2.39 | 2.19 | 2.61 | 1.74 | 1.44 | 2.10 | 2.54 | 2.08 | 3.11 | 2.69 | 2.32 | 3.12 | 2.55 | 2.12 | 3.06 |
| Worries |  |  |  |  |  |  |  |  |  |  |  |  |  |  |  |  |
|  | Financial | 1.24 | 1.15 | 1.34 | 1.16 | 1.00 | 1.35 | 1.34 | 1.14 | 1.58 | 1.42 | 1.24 | 1.64 | 1.08 | 0.91 | 1.28 |
|  | Accessing essentials | 1.24 | 1.15 | 1.34 | 1.61 | 1.38 | 1.87 | 1.11 | 0.95 | 1.30 | 1.17 | 1.02 | 1.34 | 1.14 | 0.98 | 1.34 |
|  | COVID-19 illness | 0.97 | 0.90 | 1.04 | 1.06 | 0.90 | 1.24 | 0.77 | 0.65 | 0.91 | 0.95 | 0.83 | 1.08 | 1.16 | 0.98 | 1.36 |
| Social/relationship concerns | | 1.30 | 1.20 | 1.41 | 1.57 | 1.32 | 1.85 | 1.18 | 1.00 | 1.40 | 1.33 | 1.16 | 1.53 | 1.13 | 0.94 | 1.35 |
|  | Threats to safety | 1.22 | 1.13 | 1.32 | 1.11 | 0.94 | 1.31 | 1.72 | 1.46 | 2.04 | 1.21 | 1.06 | 1.38 | 1.04 | 0.88 | 1.22 |
| Number of observations | | 63,767 | | | 7,687 | | | 18,594 | | | 23,859 | | | 13,627 | | |
|  | Number of individuals | 3,747 | | | 530 | | | 1,201 | | | 1,329 | | | 687 | | |

Note. Adversity experiences and worries variables are weighted to the proportions of gender, age, ethnicity, country, and education obtained from the Office for National Statistics. Individual adversity experiences and worries variables are binary. Analyses were further adjusted for day of the week and time since lockdown began. OR = odds ratio. CI = confidence interval.

References

1. Löwe B, Kroenke K, Herzog W, Gräfe K. Measuring depression outcome with a brief self-report instrument: sensitivity to change of the Patient Health Questionnaire (PHQ-9). J Affect Disord. 2004;81(1):61–6.

2. Spitzer RL, Kroenke K, Williams JB, Löwe B. A brief measure for assessing generalized anxiety disorder: the GAD-7. Arch Intern Med. 2006;166(10):1092–7.

3. Office for National Statistics. Population estimates for the UK, England and Wales, Scotland and Northern Ireland [Internet]. 2020 May [cited 2020 Sep 30]. Available from: https://www.ons.gov.uk/peoplepopulationandcommunity/populationandmigration/populationestimates/bulletins/annualmidyearpopulationestimates/mid2018

4. Hainmueller J, Xu Y. Ebalance: A Stata package for entropy balancing. J Stat Softw. 2013;54(7).

5. StataCorp. Stata Statistical Software: Release 16. College Station, TX: StataCorp LP; 2019.

6. Jann B. Plotting Regression Coefficients and other Estimates - Ben Jann, 2014. Stata J [Internet]. 2014 [cited 2021 Feb 3]; Available from: https://journals.sagepub.com/doi/10.1177/1536867X1401400402
